# Assessing creativity independently of language: A language-independent remote associate task (LI-RAT)

**DOI:** 10.3758/s13428-021-01773-5

**Published:** 2022-03-10

**Authors:** Maxi Becker, Roberto Cabeza

**Affiliations:** 1 Department of Psychology, Humboldt University Berlin, Unter den Linden 6, 10117 Berlin, Germany; 2 Center for Cognitive Neuroscience, Duke University, Durham, NC 27708 USA

**Keywords:** Creativity, Insight, AHA experience, Remote associate task, Creativity assessment

## Abstract

Most creativity measures are either complex or language-dependent, hindering cross-cultural creativity assessment. We have therefore developed and tested a simple, language-independent insight task based on pictures in the style of the widely used verbal remote associate task (RAT). We demonstrate that the language-independent RAT (LI-RAT) allows assessment of different aspects of insight across large samples with different languages. It also correlates with other creativity and general problem-solving tasks. The entire stimulus set, including its preliminary normative data, is made freely available. This information can be used to select items based on accuracy, mean solution time, likelihood to produce an insight, or conceptual and perceptual similarity between the pictures per item.

**Supplementary Information:**

The online version contains supplementary material available at 10.3758/s13428-021-01773-5.

## Introduction

We have developed and preliminarily normalized 121 simple pictorial remote associate problems in the style of Mednick’s remote associates task (1968). These problems are intended to extend the current set of creativity and insight tasks, which are mostly verbal, with a language-independent task. Our goal for the present article is to provide a preliminary validation of these insight problems and make them available to other researchers along with their normative data (i.e., item difficulty, likelihood for the AHA! experience including perceived suddenness upon solution) and relational properties (conceptual and perceptual similarity between the problem elements per item).

### Insight tasks and language dependency

There are different definitions of insight. Here, we will define this concept according to Danek et al. (2020) as a sudden comprehension or solution of a nonobvious problem that involves an AHA! experience (Danek et al., 2020). The AHA! experience refers to the solver’s conviction that the solution is obviously correct, emerged suddenly, and is associated with a feeling of pleasure (Danek & Wiley, 2017; Topolinski & Reber, 2010). Insight is related to creativity which, according to Mednick, is “the forming of associative elements into new combinations, which either meet specified requirements or are in some way useful” (Mednick, 1962, p. 221). More precisely, insight is a special phenomenon of creativity where a problem is solved requiring a novel solution approach that diverges from the routine (Dietrich & Kanso, 2010).

Insight problem-solving has been studied for almost 100 years with so-called classic insight problems such as the nine-dot or eight-coin problem (Köhler, 1925; Duncker, 1945; Öllinger et al., 2014). While we define the term *insight problem* as problems or tasks that are used to study insight, irrespective of whether an insight was actually elicited or not, *classic insight problems* are a specific type of insight problem. The latter usually contains a misleading problem component which has to be represented or restructured to find the solution (Weisberg, 1995). However, researchers using classic insight problems face theoretical and methodological difficulties: First, those problems are usually complex, allowing participants to solve only few items in one experiment. Using only few problems reduces the reliability of the data collected. Additionally, complex problems make it more difficult to disentangle different component processes during the solution process (Bowden & Jung-Beeman, 2003a). Finally, it was assumed that a correct solution of those problems would always elicit an insight, i.e., an AHA! experience, but this view has been challenged (Danek et al., 2016; Webb et al., 2016).

For these reasons, researchers have developed simpler insight tasks specifically using participant’s subjective AHA! self-reports instead of assuming that insight was elicited (see Bowden & Grunewald, 2018, for discussion). Such simpler insight tasks are the remote associates task (RAT, Mednick, 1962) or different versions of it like the compound remote associate task^1^ (C-RAT, see Bowden & Jung-Beeman, 2003a). A remote associate problem consists of three cue words (“fox,” “man,” and “peep”), and participants are required to find the solution word (“hole”) that is associated to all three cue words in a number of ways.

The RAT/C-RAT reflects creative cognition because solvers need to think of more distantly related lexical-semantic information in order to relate the three cue words. In addition, accuracy in this task reliably correlates with accuracy in classic insight problems (Schooler & Melcher, 1995). The RAT/C-RAT has been widely used to investigate insight and its component processes behaviorally (e.g., Kizilirmak et al., 2016a; Bowden & Beeman, 1998; Webb et al., 2016; Cunningham et al., 2009) and neurocognitively (Jung-Beeman et al., 2004; Kounios et al., 2006; Kizilirmak et al., 2016b; Luft et al., 2018; Becker et al., 2020b, c).

One limitation of the RAT/C-RAT is that it is language-dependent. That is to say, the solver must have knowledge of the specific language in which the problem elements (cue words) are presented to solve the task. To address this limitation, researchers have translated and re-normed the original English version to different languages, including Dutch (Chermahini et al., 2012), Chinese (Wu & Chen, 2017), Japanese (Baba, 1982), or German (Landmann et al., 2014; Becker et al., 2020a). Unfortunately, the translated versions are not identical, often using different items. Thus, although differences in performance between different language samples have been reported (Behrens & Olteteanu, 2020), it is unknown if these differences reflect differences in the populations’ ability to solve the problems or in the problem difficulty due to the different language (Behrens & Olteteanu, 2020). As a result, it is difficult to use RAT/C-RAT to investigate cultural differences in creativity and insight. In addition to cross-culture comparisons, the language-dependent RAT is not ideal to evaluate creativity abilities in individuals with a limited vocabulary, including illiterate and uneducated populations, as well as immigrants with partial knowledge of the language. Finally, the language-dependent RAT task cannot be used to investigate creativity and insight in patients with language deficits due to brain lesions or degenerative diseases (de Souza et al., 2010; see review Palmiero et al., 2012).

There are a few other language-independent creativity tasks, but they are either limited in their number of items, simplicity, easiness of their application, or their availability. For example, the figural component of the Torrance Test of Creative Thinking (TTCT, Torrance, 1966) and the Test of Creative Imagery Abilities (TCIA, Jankowska & Karwowski, 2015) are both language-independent and validated. However, they require drawing, contain few items, and in particular for the TTCT their application and evaluation is labor-intensive and requires trained personnel (Kim, 2006; Swartz, 1988; Jankowska & Karwowski, 2015).

In sum, a language-independent remote associate test is needed in the creativity domain to investigate cross-cultural differences, populations of reduced vocabulary (e.g., illiterate, uneducated, and language-challenged immigrants), and brain disorders that result in reduced linguistic abilities. Aiming to fill this gap and to extend the current set of RATs, we developed a language-independent RAT (henceforth LI-RAT) and provided a preliminary validation for it. The individual items, an Inquisit script to run the study online (see GitHub: https://github.com/MaxiBecker/LI-RAT.git) and preliminary normative data for three language samples (see Appendix, Tables S1–S2), are freely available.

### The LI-RAT and the current studies

**Fig. 1:**
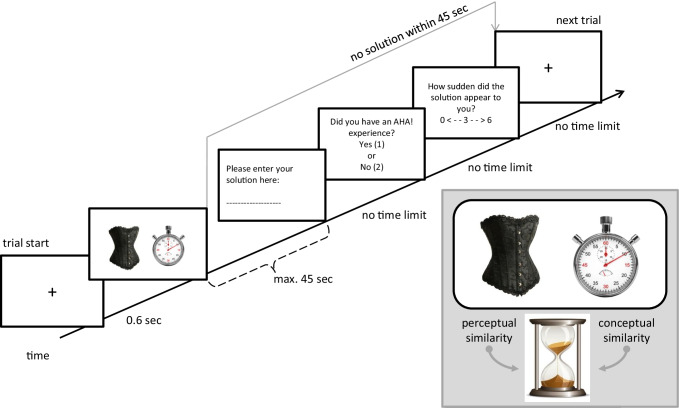
Timeline of the LI-RAT paradigm and example item. *Note*. Participants are asked to find an object (hourglass) that connects the two seemingly unrelated presented objects (corset, stopwatch) within max. 45 s. They are told that the solution is conceptually but not perceptually related to the stopwatch and perceptually but not conceptually related to the corset (see right panel). Upon solution, they are requested to rate their AHA! experience (including how sudden the solution appeared to them; see left panel).

The LI-RAT is constructed in a similar way as Mednick’s (1962) original version of the (verbal) remote associates, relying on participant’s subjective AHA! self-reports. However, instead of three words, participants receive two object pictures (cues), such as the pictures of a corset and a stopwatch, and they have to find a third object (target) that can be connected to both cue pictures, such as hourglass (see Fig. 1). As illustrated by this example, the target object can be connected to the cue objects perceptually (e.g., the corset and the hourglass have a similar shape) or conceptually (e.g., both the stopwatch and the hourglass measure time) (see Method’s section for further details). This task taps creative insight processes because solving each problem requires thinking of everyday objects in an unusual way. For example, to find the relationship between corset and hourglass, the solver needs to completely ignore the dominant conceptual and functional representation of a corset as a garment, and focus on its shape. Different problems emphasize different perceptual aspects (shape, color, etc.), but in general, one of the objects has to be processed purely perceptually.

In the current article, we report two studies preliminarily norming the new LI-RAT and investigating it in relation to other creativity and cognitive measures. The first study was used to create normed information on the LI-RAT items regarding their difficulty and likelihood to elicit insight. Data were acquired and compared between three different languages: English, German, and Spanish. In the second study, we provide a preliminary external validation of the LI-RAT by investigating to what degree it shares common variance with two widely used creativity tasks, the C-RAT and Alternative Uses Task (AUT), as well as with general problem-solving ability, as measured by the Raven’s advance progressive matrices test (henceforth Raven’s matrices).

## Study 1

The goal of this study was twofold. First, we preliminarily normed the individual items of the LI-RAT in terms of difficulty and ability to elicit an insight for three different language samples (English, German, and Spanish). Second, we tested the hypothesis that the LI-RAT is language-independent. We assumed that if the LI-RAT were language-independent, then its items should exhibit a comparable distribution and correlate between all three samples in variables of interest: performance (accuracy, solution time) and self-reported AHA! experience (including perceived suddenness of the solution). To further characterize this task for exploratory reasons, we provide additional information on the impact of demographic variables (age, gender) and verbal semantic fluency on LI-RAT performance and likelihood to produce an insight.

### Methods

#### Construction of the LI-RAT

Participants receive two pictures (cues) and need to find a third object (*target*) that is related to the two cues. One of the cues (*conceptual cue*) is always conceptually similar but visually dissimilar to the target, and the other cue (*visual cue*) is visually similar but conceptually dissimilar to the target. For example, participants are presented with a picture of a corset and a stopwatch (see Fig. 1). The solution is an hourglass because its shape is visually similar to the hyperboloid shape of the corset, and it is conceptually similar to the stopwatch (both objects measure time).

The visual cue (e.g., corset) requires thinking of a real object in an unusual way, focusing on its visual features (shape, color, etc.) rather than its function or meaning, which is the standard way in which we categorize objects in everyday life. This is the key creative component of the test because it requires going beyond standard categories or schemas. Having one visual cue and one conceptual cue also help constrain the solution to one main solution (target: hourglass), although some of the problems can have alternative solutions (see Tables S1–S2 in the Appendix).

To avoid potential priming effects, solution words were never repeated or used as cue objects. Also, cue objects did not repeat with a few exceptions (see Appendix, Tables S1–S2). The preliminary normative data on performance (accuracy, solution time) and aspects of the AHA! experience (including suddenness of the solution) is provided in the Appendix.

A total of 141 LI-RAT items were created by three subjects (M.B. and two lab members), based on the rules mentioned above (one cue was visually similar but conceptually dissimilar to the target and vice versa for the other cue). Visual similarity was established based on general shape, a specific feature (e.g., trunk of an elephant and neck of a watering can) or a specific combination of colors (black/white of a nun and a panda bear). Conceptual similarity was established when both objects belonged to the same category (e.g*., frog & snail -> amphibian*), or they were associatively related by occurring in a similar context (e.g., *bulb & sun* -> *light*). Additionally, we aimed to find cues that would be as dissimilar to each other as possible. Further constraints were that the cues (and the target) represented concrete, common objects with a definable shape (e.g., *swimming pool* instead of *water*) that can be displayed as a picture on a white background. The created items were cross-checked by the other two lab members to see whether they met the abovementioned criteria. More importantly, the 141 items were piloted iteratively online via Mechanical Turk based on a sample of 10 English speaking subjects according to their accuracy. Subsequently, we selected a subset of those items that had an item difficulty of at least 35%^2^. This resulted in a total of 121 LI-RAT items. Performance and different aspects of the AHA! experience were validated based on an English- and German-speaking sample in study 1. Finally, the relationship to other creativity and problem-solving tasks was investigated in study 2.

Both studies were preregistered at https://aspredicted.org/blind.php?x=s2n4cb; preregistration adheres to the disclosure requirements of the institutional registry (note, the preregistration for study 1 does not include the Spanish language sample as it was added later). The pictures that refer to the 121 validated LI-RAT items as well as the Inquisit script to run the task online and offline are freely available on GitHub (https://github.com/MaxiBecker/LI-RAT.git).

#### Confirming the relationships of the target to visual and conceptual cues

To confirm the assumptions made when creating the test, we measured visual similarity and conceptual similarity between the solution target and the two cues. We expected that visual similarity between the solution and the cues would be stronger for the visual cues than for the conceptual cues and that the conceptual similarity between the target and the cues would be stronger for the conceptual cues than for the visual cues.

We quantified the visual similarity using the penultimate convolutional layer of a pretrained convolutional neural network called AlexNet (Krizhevsky et al., 2012). Deep neural networks such as AlexNet are becoming increasingly popular in visual neuroscience (Gauthier & Tarr, 2016; Kriegeskorte, 2015; Davis et al., 2021). AlexNet was trained on more than a million pictures of the ImageNet database (http://www.image-net.org). Visual similarity between two objects was quantified as the Spearman correlation between each cell in the penultimate (fully connected) layer of AlexNet (for more details, see Davis et al., 2021). On average, visual similarity between targets and cues was significantly greater (*t*(121) = 2.96, *p* < .01, mean difference = .072, *CI* = [0.02–0.12]) for visual cues (*M* = 0.61, *SD* = 0.20) than for conceptual cues (*M* = 0.53, *SD* = 0.20).

The conceptual similarity of the cues to the solution was quantified via cosine distances derived from a preexisting word-embedding model: 72 million well-formed sentences from the Leipizig Corpora Collection sampled from news websites in German language (Biemann et al., 2007) as well as additional 1.4 million sentences from web texts (for more in details, see Becker et al., 2020a; see also Levy & Goldberg, 2014; Mikolov et al., 2013). The database for the current word-embedding model consisted of consists of 455,050 words represented each in a 300-dimensional vector. The cosine similarity between a target and a cue describes the angle between the two respective (300-dimensional) vectors in this word space. As expected, conceptual similarity between targets and cues was significantly greater (*t*(121) = 4.13, *p* < .001, mean difference = .054, *CI* = [0.03–0.08]) for conceptual cues (*M* = 0.46, *SD* = 0.13) than for visual cues (*M* = 0.41, *SD* = 0.09).

#### Participants

We originally planned a comparison only between two language samples (English, German). Using G*power (Faul et al. 2007), we estimated that we would need at least a total sample size of *n* = 99 to show a significant between-sample correlation for the LI-RAT items (assuming a minimal effect size |ρ| = .3, α = .05, β = .80, 20% dropout rate). However, we recruited more than 50 subjects per sample to increase the accuracy of the preliminarily normed items per sample. The original English-speaking sample consisted of 183 participants recruited via the online platform Mechanical Turk. Due to low performance (less than 10% correct: *n* = 8) or premature termination of the task (*n* = 20), we excluded 28 subjects (~15%) which resulted in a final sample of 155 English-speaking participants [age (in years): range= 25–69; 73 females: *M* = 44.0; 82 males: *M* = 38.3]. The German-speaking sample consisted of 66 participants recruited via an online student platform in Hamburg and Berlin. Due to too low performance (less than 10% correct: *n* = 1) or premature termination of the task (*n* = 5), we excluded six subjects (~9%) which resulted in a final sample of 60 German-speaking participants [age (in years): range = 20–62; 38 females: *M* = 29.6; 21 males: *M* = 29.7] (four participants did not indicate their gender). Note, not excluding subjects due to too low performance did not significantly change the results in either the English or the German sample.

To further cross-culturally compare the results of the German- and English-speaking sample, we additionally recruited a Spanish-speaking sample (*n* = 65) via the online platform Prolific (https://prolific.co/). For better comparability, we recruited the same amount of subjects in the Spanish as in the German sample. Due to premature termination of the task, we excluded a total three participants (~5%) which resulted in a final sample of *n* = 62 [age (in years): range = 18–50; 34 females: *M* = 27.5; 28 males: *M* = 26.4]. Informed consent was obtained from all individual participants included in the study, and they received a monetary compensation according to their time on task. The local ethics committees of Humboldt University and Duke University approved of the study.

#### Materials and procedure

All three samples received 61 randomly chosen LI-RAT items from a pool of 121 pre-validated items (see section *Construction of LI-RATs* in the *Introduction*; the complete list of items can be viewed in the Appendix). The participants were tested individually online via the research software Inquisit 4.0 (Draine, 1998). The participants were instructed (in their respective native language) to find an object [hourglass] that was conceptually similar (but visually dissimilar) to one object [stopwatch] on the screen and visually similar (but conceptually dissimilar) to the other object [corset] on the screen. Participants completed two practice trials before starting the experiment.

The timeline of the experiment is depicted in Fig. 1. The trial started with a fixation cross for 600 ms followed by the stimulus onset of two pictures [e.g., corset and stopwatch] on a white background. The dimension of the pictures was 488 × 488 pixels. The participants were instructed to press Enter when they were convinced of having found the correct solution. The two pictures were presented until the Enter button was pressed or for a maximum of 45 seconds (time out). If the participants did not find a solution within 45 seconds, the next trial would start. If participants responded within the allotted time, they were instructed to type in their solution, that is, the name of the object they thought of (no time limit). Subsequently, participants were asked to judge how they experienced their respective solution. Prior research has shown that the AHA! experience itself can be split into different aspects: certainty about the correctness of the solution, how sudden the solution appears to the solver, and the positive emotional response or pleasure upon the solution (Danek & Wiley, 2017). Here, we concentrated on the pleasure upon and the perceived suddenness of the solution, which were described to the participants as follows:

The AHA! experience is the feeling of pleasure when the solution came to you in a sudden manner. This can also be the case when you have already searched for the solution for quite some time. In contrast, the solution without insight (AHA! experience) appears to you in a more step-wise manner. For example, through active search you feel like you increasingly approached the solution.

The participants were first asked to rate whether they experienced an AHA! (yes/no answer). Because the AHA! experience is more strongly focused on the emotional response given the definition, we additionally asked participants to rate the perceived suddenness of the solution on a scale from 0 to 6. There was no time limit for both responses. Subsequently, a new trial would start.

##### Verbal semantic fluency

We were additionally interested in whether verbal semantic fluency could explain variance in the LI-RAT as has been shown for other creativity tasks such as the AUT (Silvia et al., 2013; Forthmann et al., 2019). In the LI-RAT, subjects constantly have to produce potential solution words related to the category of the conceptually similar target object. Verbal fluency tasks have been shown to quantify lexical retrieval ability (see Federmeier et al., 2002, 2010; Cohen et al., 1999). For this reason, we assessed semantic fluency in a short online task (Benton, 1968). Participants were given 90 seconds to type into the computer as many unique words as possible within a semantic category. The two categories were “animals” and “plants.” The variable of interest was the number of correctly named words summed up over both categories. Average split half reliability of this measure over all three samples was λ = .71 (Guttman, 1945).

#### Analysis

##### Normative data

Tables S1–S2 in the Appendix display the main measures for each individual LI-RAT item for the English, German, and Spanish sample separately. These measures are the mean and standard deviation (SD) for (1) accuracy (probability of solving each problem), (2) solution time (in general and when the item was solved correctly), and (3) the AHA! experience as well as the (4) perceived suddenness of the solution. We additionally added both measures of perceptual and conceptual similarity between the cues to the solution for every item (see Table S1, Appendix).

##### Rules to determine accuracy

An answer was counted correctly if the named object fulfilled the following task constraints: The solution object must be visually similar (but conceptually dissimilar) to the object in one of the two shown pictures and conceptually similar (but visually dissimilar) to the object in the other shown picture. Visual similarity was given if two objects shared a) the same overall shape, b) one specific feature (for example, the trunk of an elephant and an unusually long shaped neck of a watering can), or c) a combination of colors like black and white (a shared single color did not count as visual similarity). Conceptual similarity was given if two objects belong either to the same category (e.g., *frog & snail -> amphibian*) or if they are associatively related by occurring in a similar context (e.g., *bus & ticket* -> *travel* or *bulb & sun* –> *light*).

Participants were instructed to follow those similarity rules. We specifically designed the task to avoid several possible solutions, but in roughly one fourth of the cases (32% English; 21% German, 31% Spanish), at least one subject still found at least one correct alternative solution (see Appendix, Tables S1–S2, *amount N with alternative solutions*). In those cases, the solution was still counted as correct. The range of different alternative solutions per problem ranged from 0 to 4. We marked those items in the Appendix (Tables S1–S2) with an asterisk that had three or four correct alternative solutions in the respective language.

##### Item-level comparison of performance & AHA! experience between samples

**Fig. 2:**
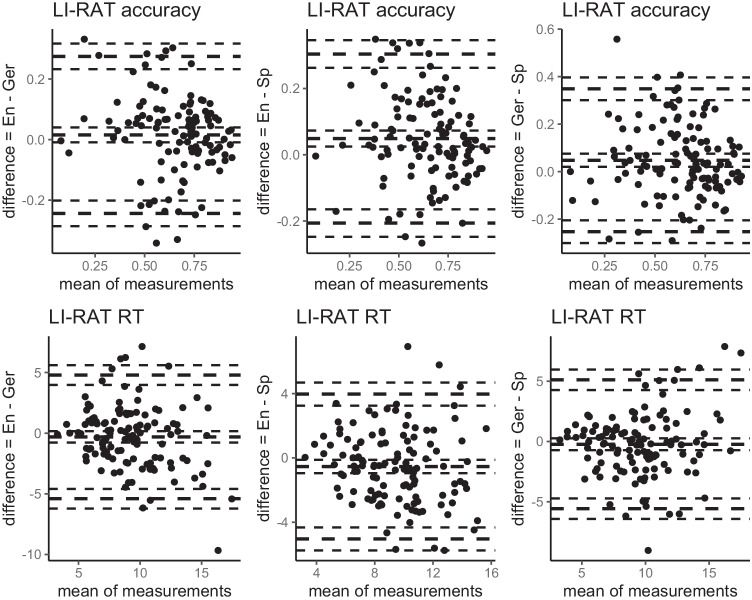
Bland–Altman Plots for performance measures of LI-RAT comparing between English, German, and Spanish samples (item level). *Note*. RT = solution time; En = English sample; Ger = German sample; Sp = Spanish sample. The outer thick lines represent the 1.96 standard deviation from the mean (middle thick line). The thinner lines around the thick lines represent the respective 95% confidence interval.

**Fig. 3:**
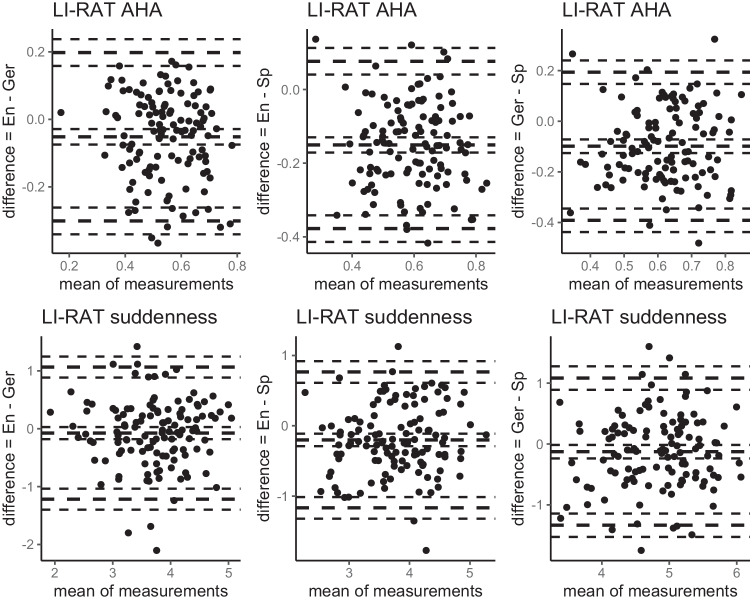
Bland–Altman plots for performance measures of LI-RAT comparing between English, German, and Spanish samples (item level). *Note*. En = English sample; Ger = German sample; Sp = Spanish sample. The outer thick lines represent the 1.96 standard deviation from the mean (middle thick line). The thinner lines around the thick lines represent the respective 95% confidence interval.

To show that the LI-RAT is comparable across samples, we correlated accuracy, solution time, and the AHA! experience including suddenness for all items between all three samples using Pearson’s correlations. All 95% confidence intervals were bootstrapped using the *spearmanRho* function (version 2.3.26) in R (10,000 replications). The level of significance was set to *p* < .05. To further visually compare the LI-RAT items between the samples, we provided Bland–Altman plots in Figs. 2 and 3 for accuracy, solution time, AHA! experience, and the perceived suddenness of the solution (see Giavarina, 2015). Finally, absolute values in terms of item-wise means and standard deviations for performance and AHA! experience measures between all three samples are provided. To quantify the magnitude of the difference in absolute values for item difficulty (accuracy, solution time) and AHA! experience including suddenness between all three language samples, Cohen’s *d* and two-sample *t*-tests were used. *P*-values and confidence intervals were based on 10,000 permutations using the MKInfer package (version 0.6) in R (R Core Team, 2014).

##### Subject-level comparison of performance & AHA! experience between samples

To further characterize the LI-RAT for exploratory reasons, we assessed whether LI-RAT scores vary across language samples, demographics (gender and age), and verbal fluency. To investigate this, we set up four different general linear models (GLMs). Sample (English, German, or Spanish), age (in years), gender (binary variable), and verbal fluency (continuous count variable) served as independent variables. Accuracy, solution time, and the AHA! experience including perceived suddenness of the solution served as dependent variables. To reach normality of the error distribution, solution time was log-transformed. Solution time and suddenness were modeled using the Gaussian identity link function. Accuracy and the self-reported AHA! experience upon solution were modeled using a beta regression because both variables are continuous but restricted to the unit interval (0,1) (Ferrari & Cribari-Neto, 2004; Cribari-Neto & Zeileis, 2010). Standardized coefficients and 95% confidence intervals for the individual predictors per GLM as an effect size measure were calculated using the R package *sjplot* (version 2.8.7; see Lüdecke, 2018). The level of significance for all statistical tests was set to *p* < .05.

### Results and Discussion

The item-wise means and standard deviations (SDs) of the two performance measures, accuracy and solution time, and the AHA! experience including perceived suddenness of the solution are reported in the Appendix (Tables S1–S2) separately for all three samples.

#### Performance: item level

First, we investigated differences in absolute item difficulty between all three languages. On average, item difficulty was 66.3% (*SD* = 18.0%) in the English sample, 64.9% (*SD* = 19.8%) in the German sample, and 60.1% (*SD* = 21.0%) in the Spanish sample. Accuracy for items in the English and German (*t*(238.1) = .60, *p* > .05, Cohen’s *d* = 0.08) and the German and Spanish sample (*t*(238.1) = 1.82, *p* > .05, Cohen’s *d* = 0.23) did not differ significantly, but they differed between the English and Spanish sample (*t*(238.1) = 2.47, *p* < .05, Cohen’s *d* = 0.33). When removing items (*n* = 18) whose difference in accuracy between the English and Spanish sample was greater than 1.5 SD from the mean, the overall difference in item difficulty between both samples disappeared (*t*(202.5) = 1.82 *p* > .05, Cohen’s *d* = .17; those 18 items are marked with a double dagger (‡) in Table S2 in the Appendix). Importantly, all three samples were highly comparable in terms of relative item difficulty: the accuracy of all LI-RAT items correlated significantly between the English and German sample (*r*_*p*_ = .71, *p* < .05, *CI* = [.56–.82]), between the English and Spanish sample (*r*_*p*_ = .69, *p* < .05, *CI* = [.59–.78]), and between the German and Spanish sample (*r*_*p*_ = .72, *p* < .05, *CI* = [.62–.79]).

Furthermore, average solution time was 9.1 s (*SD* = 2.8 s) in the English sample, 9.4 s (*SD* = 3.4 s) in the German sample, and 9.7 s (*SD* = 3.1 s) in the Spanish sample. There was no evidence that solution time differed between the three samples (English–German, *t*(230.7) = −.76, *p* > .05, Cohen’s *d* = 0.10; English–Spanish, *t*(237.55) = −1.41, *p* > .05, Cohen’s *d* = 0.18; and German–Spanish, *t*(237.55) = −.55, *p* > .05, Cohen’s *d* = 0.07). Average solution time of the LI-RAT items correlated significantly between the English and German sample (*r*_*p*_ = .68, *p* < .05, *CI* = [.57–.77]), between the English and Spanish sample (*r*_*p*_ = .72, *p* < .05, *CI* = [.62–.80]), and between the German and Spanish sample (*r*_*p*_ = .66, *p* < .05, *CI* = [.54–.75]). Figure 2 shows Bland–Altman plots to visually compare performance measures between all three language samples.

#### Performance: subject level

Average accuracy was 66.5% (*SD* = 14.6%) for the English-speaking sample, 65.1% (*SD* = 15.2%) for the German-speaking sample, and 63.5% (*SD* = 18.3%) for the Spanish-speaking sample. Those differences were not significant (*p* > .1), but when controlling for age, sex, and word fluency, accuracy was different between the Spanish and German sample (*ß* = −.31, *CI* = [−.57 to −.04]), while there was still no significant difference between the English and the German sample (*ß* = .13, *CI* = [−.13–.39]) or between the English and the Spanish sample (*ß* = −.18, *CI* = [−.40–.05]). Differences in absolute accuracy values in the German and Spanish sample could be due to general differences (e.g., education, etc.) between both drawn samples (note, both samples were recruited via different online sources [Spanish: Prolific; German: Stellenwerk.de]). More importantly, relative accuracy in terms of between-item correlations between both samples was still highly comparable (see above, *r*_*p*_ = .72). Furthermore, accuracy was significantly predicted by word fluency (*ß* = .17, *CI* = [.09–.25]) but not by gender (*ß* = −.06, *CI* = [−.22–.11]) or age (*ß* = .00, *CI* = [−.10–.10]). Table S3 in the Appendix summarizes the results from the GLM predicting accuracy.

Additionally, average solution time was comparable between all three samples (English-speaking sample: 9.3 s [*SD* = 3.2 s]; German-speaking sample: 9.6 s, [*SD* = 3.6 s]; Spanish-speaking sample: 9.8 s [SD = 2.9 s]). There was no significant difference in solution time between the English and the German sample (ß = −.01, *CI* = [−.14–.11]), English and Spanish sample (ß = .08, *CI* = [−.02–.19]), or German and Spanish sample (ß = .10, *CI* = [−.03–.22]). Furthermore, consistent with the results on accuracy, word fluency (ß = −.12, CI = [−.16 to −.08]) negatively predicted solution time in the LI-RAT. The observed relationship between the LI-RAT and word fluency may relate to an overall ability to fluently produce possible solutions given a set of constraints (see also General Discussion). There was no evidence for an effect of gender (ß = .03, *CI* = [−.05–.11]). However, age (ß = .07, *CI* = [.02–.11]) positively predicted solution time, consistent with age-related slowing (Salthouse, 1996). Table S3 in the Appendix summarizes the results from the GLM predicting solution time.

#### AHA! experience: item level

On average, solving the LI-RAT elicited an AHA! experience in 51.8% (*SD* = 12.2%) of all items in the English sample, 57.0% (*SD* = 13.5%) in the German sample, and 66.9% (*SD* = 12.8%) in the Spanish sample. The subjective AHA! experience significantly differed between all three languages for the LI-RAT items (English–German *t*(237.8) = −3.11, *p* < .05, Cohen’s *d* = 0.40; English–Spanish *t*(239.58) = −9.35, *p* < .05, Cohen’s *d* = 1.21; German–Spanish *t*(239.3) = −5.86, *p* < .05, Cohen’s *d* = 0.75). As the Bland–Altman plots demonstrate, it was not the outliers driving this difference, but the mean was systematically shifted, especially between the Spanish and the other two language samples. Prior studies have demonstrated that there are cultural differences in frequency and intensity of positive emotional responses (Lim, 2016; Lewis et al., 2010). Compared to European American and other cultural groups, Hispanics show a specifically high amount of positive emotions (Scollon et al., 2004).

Importantly, however, even though the absolute values in AHA! experience differed between the language samples, there was still a significant relationship between the items: The AHA! experience of the LI-RAT items correlated significantly between the English and German sample (*r*_*p*_ = .51, *p* < .05, *CI* = [.39–.63]), between the English and Spanish sample (*r*_*p*_ = .57, *p* < .05, *CI* = [.47–.66]), and (to a lesser extent) between the German and Spanish sample (*r*_*p*_ = .36, *p* < .05, *CI* = [.22–.48]).

Furthermore, experienced suddenness upon solving the LI-RAT items (on a scale between 0 and 6) was rated with 3.7 (*SD* = 0.7) in the English sample, 3.8 (*SD* = 0.7) in the German sample, and 3.9 (*SD* = 0.7) in the Spanish sample. Rated suddenness did not differ between the English and German sample (*t*(240) = −.83, *p* > .05, Cohen’s *d* = .11) or between the German and Spanish sample (*t*(238.9) = −1.43, *p* > .05, Cohen’s *d* = .18), but it differed between the English and Spanish sample (*t*(239.1) = −2.30, *p* < .05, Cohen’s *d* = .29). When removing items (*n* = 3) whose difference in suddenness between the English and Spanish sample was greater than 2 SD from the mean, the overall difference in item difficulty between both samples disappeared (*t*(232.1) = −1.32 *p* > .05, Cohen’s *d* = .17; those three items are marked with an asterisk in Table S2 in the Appendix, first column). Importantly, average experienced suddenness upon solution of the LI-RAT items correlated significantly between the English and German sample (*r*_*p*_ = .65, *p* < .05, *CI* = [.52–.76]), between the English and Spanish sample (*r*_*p*_ = .74, *p* < .05, *CI* = [.65–.81]), and between the German and Spanish sample (*r*_*p*_ = .59, *p* < .05, *CI* = [.47–.70]). Figure 3 shows Bland–Altman plots to visually compare the AHA! experience including suddenness between all three language samples.

#### AHA! experience: subject level

On average, the AHA! experience was reported for 52.3% (*SD* = 23.4%) of the solved problems in the English-speaking sample, for 56.6% (*SD* = 20.4%) of the solved problems in the German-speaking sample, and for 67.1% (*SD* = 19.0%) of the solved problems in the Spanish-speaking sample (note, the AHA! experience was measured in a binary manner, hence as present or absent; see Bowden & Jung-Beeman, 2003a). While the self-reported AHA! experience between the English and German sample was not significantly different (*ß* = .33, *CI* = [−.03–.69]), the English and the Spanish sample (*ß* = .73, *CI* = [.41–1.05]) and the German and the Spanish sample (*ß* = .40, *CI* = [.03–.77]) differed significantly in their self-reported AHA! experience. Neither word fluency (*ß* = .05, *CI* = [−.07–.16]) nor gender (*ß* = −.00, *CI* = [−.23–.23]) nor age (ß = .07, *CI =* [−.06–.21]) predicted the AHA! experience. Table S3 in the Appendix summarizes the results from the GLM predicting the AHA! experience.

The average perceived suddenness of the solutions (0 = solution appeared in a continuous manner to 6 = solution appeared in a very sudden manner) was 3.7 (*SD* = 0.90) in the English-speaking participants, 3.8 (*SD* = 0.81) in German-speaking participants, and 3.9 (*SD* = 0.61) in the Spanish-speaking participants. There was no evidence for a difference in perceived suddenness of the solution between the English and German (*ß* = .31, *CI* = [−.10–.73]), English and Spanish (*ß* = .29, *CI* = [−.06–.65]), or German and Spanish sample (*ß* = −.02, *CI* = [−.44–.40]). Neither word fluency (*ß* = .07, *CI* = [−.05–.20]) nor age (ß = .01, *CI* = [−.14–.16]) predicted suddenness, but gender (*ß* = −.27, *CI* = [−.53 to −.01]) did. Female participants experienced the solution as less sudden. Although gender effects in verbal ability are well documented (but also critically discussed; see meta-analysis of Hyde & Linn, 1988), gender differences in insight have not been reported before. Further research is required to investigate why gender effects one (suddenness) of the two components of insight. Table S3 in the Appendix summarizes the results from the GLM predicting perceived suddenness.

**Fig. 4:**
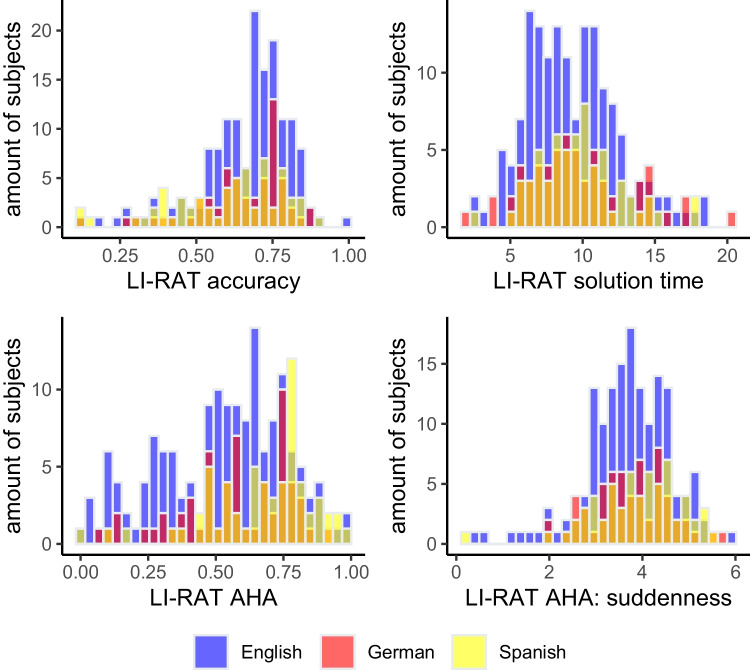
Distribution of average performance and AHA! experience in the LI-RAT for an English-, German-, and Spanish-speaking sample (subject level). *Note*. The English-speaking sample included 155 participants, whereas the German-speaking sample only included 60 participants, and the Spanish 62 participants. The values represent the average performance and average AHA! experience including suddenness per person.

Figure 4 displays histograms for the averaged values of accuracy, solution time, the AHA! experience, and additionally suddenness for both samples (Figure S7 in the appendix additionally depicts the raw values for the AHA! experience and suddenness).

## Study 2

The goal of the second study was to provide a preliminary validation for the LI-RAT. For this, we investigated to what degree it shares common variance with two widely used creativity tasks, the C-RAT and Alternative Uses Task (AUT), as well as with general problem-solving ability, as measured by the Raven’s matrices.

Given that both the LI-RAT and the C-RAT involve finding remote associations, we expected they will be significantly correlated. In contrast, the relationship between the LI-RAT and the AUT was difficult to predict. One study found a significant correlation between the RAT and the AUT (Salvi et al., 2020), but another study did not (Chermahini et al., 2012). We assumed that compared to the RAT, the LI-RAT would have a stronger association with the AUT because both tasks involve imagining a concrete object in a different way than its standard function (e.g., a corset as a shape similar to an hourglass rather than as a garment). Finally, there have been various accounts linking insight problem-solving to the general ability to solve problems as measured by Raven’s matrices (Ash & Wiley, 2006; Raven et al., 1983; Gilhooly & Fioratou, 2009; Chuderski & Jastrzębski, 2018). Given this evidence, we expected a significant correlation between the LI-RAT and the Raven’s matrices.

### Methods

#### Participants

To preliminarily validate the LI-RAT with other creativity and general problem-solving measures, we estimated a necessary sample size of at least 67 subjects to detect a meaningful correlation of r = .3 (α = .05; β = .80). Assuming a 20% dropout rate (incomplete data sets or too low performance in either of the tasks), we ended up testing 79 participants. Due to premature termination of the experiment (*n* = 9), we excluded nine subjects which resulted in a final sample of 70 participants [age (in years) range = 18–39; 48 females: *M* = 25.4; 20 males: *M* = 28.4; note, two participants did not indicate their gender]. The participants were recruited via an online student platform in Hamburg and Berlin. For this study, we only included participants that reported to be German native speakers due to the compound remote associate test which was in German language. The local ethics committee of Humboldt University approved of the study. All participants received a monetary compensation, and informed consent was obtained from all individuals included in the study.

#### Materials and procedure

The participants received an online test battery including the LI-RAT, the compound remote associate task, the AUT, and Raven’s advanced progressive matrices (in this order). The test battery was presented online via Inquisit 4.0 (Draine, 1998).

##### LI-RAT

The test including the procedure and timing was identical to the one described in study 1 with the difference that only 20 items were presented to the participants instead of 61. The items were chosen such that difficulty would be uniformly distributed (i.e., the same amount of simple, medium, and difficult items). The main variable of interest was accuracy, hence the number of correctly solved problems as well as the average AHA! experience and the amount of perceived suddenness of the solution. This measure showed good internal consistency (α = .78; *CI* = [.72–.85]; λ = .79). We applied the same rules to determine accuracy as in study 1. For exploratory purposes and to exclude variance due to a speed/accuracy tradeoff, we additionally computed a quotient of accuracy divided by the average solution time.

##### Compound remote associates

The task included 20 German compound remote associate problems^3^ of varying difficulty as described and verified in a previous study (Becker et al., 2020a; for the original English version of this task, see Bowden & Jung-Beeman, 2003a). Participants were asked to find a compound word that meaningfully combines three presented words. For example, participants were presented with the words “*drop,*” “*coat,*” and “*summer,*” and they needed to find the solution “*rain*” (“raindrop,” “raincoat,” or “summer rain”). Except for the different stimulus presentation (three words instead of two pictures), the procedure and timing were exactly identical to the LI-RAT. The main variable of interest was accuracy, hence the amount of correctly solved problems as well as the average AHA! experience and the amount of perceived suddenness of the solution. This measure showed good internal consistency and split half reliability (α = .78; *CI* = [.72–.85]; λ = .79). For exploratory purposes, we additionally computed a quotient of accuracy divided by the average solution time to control for a speed/accuracy tradeoff.

##### Alternative uses task

Participants were asked to enter as many creative uses as they can think of for three target objects: newspaper, brick, and shoe into fixed text boxes on the screen. The time limit for each target object was 90 seconds. Each participant’s output was scored for (1) *fluency*, (2) *flexibility*, and (3) *originality* per target object and subsequently averaged over all three objects. *Fluency* comprises the amount of correctly named uses per object. Note, a correct use per object is a use that “should be possible for the object” (Guilford et al., 1960, p. 30). An average fluency score was generated in response to the three objects for each participant. *Flexibility* describes the amount of different conceptual categories that the generated responses could be allocated to (Guilford, 1967). The amount of different conceptual categories generated per object was averaged for all three objects for each participant. For *originality,* we used a method of uniqueness scoring (see Torrance, 1974; Runco, 2008). The uniqueness of a response was based on the probability of its occurrence within a sample. A given response received a zero unless it occurred only in 5% or 1% of all responses. In this case, it was assigned with one or two points, respectively. All numbers were summed up to generate a total uniqueness score for each participant. *Flexibility* scorings were conducted by two independent experienced raters (average age = 29.0 years which is comparable to the mean age of the sample, *M* = 26.4). Inter-rater reliability was high (*r*_p_ = .94, *CI* = [.91–.96]). *Fluency* (α = .77, *CI* = [.68–.87]; λ = .85) and to a lesser extent *flexibility* (α = .56, *CI* = [.37–.74]; λ = .69) showed an acceptable internal consistency and average split half reliability. However, the measure *originality* was less reliable (α = .38, *CI* = [.13–.63], λ = .48) (for a discussion on reliability of this sub-score, see Benedek et al., 2013).

##### Raven’s advanced progressive matrices

Language-independent general problem-solving ability was quantified via Raven’s matrices (Raven et al., 1983). The task’s problems consisted of a three-by-three matrix of figural patterns, while the bottom-right pattern was missing. The goal was to find the correct missing pattern by choosing one of the eight response options, each comprising the patterns that could match the missing one.

To keep the test as short as possible but still reliable, we used the validated short version including only 12 items (Arthur & Day, 1994). Participants had 100 seconds to complete one trial. The main variable of interest in this task was accuracy quantified as the amount of correctly solved items. This measure showed acceptable reliability (α = .68; *CI* = [.57–.79], λ = .67).

For exploratory purposes, we additionally computed a quotient of accuracy divided by the total amount of time of task to control for a speed accuracy trade-off.

##### Analysis

To analyze the relationship between variables of interest (performance parameters and the two aspects of the AHA! experience) in the LI-RAT and other creativity and problem-solving tasks, we computed Spearman’s rank coefficient (*r*_s_). This correlation method is more robust towards violations of the normal distribution and outliers compared to Pearson’s product moment correlation coefficient (de Winter et al., 2016). All 95% confidence intervals were bootstrapped using the *spearmanRho* function (version 2.3.26) in R (10,000 replications). The level of significance was set to *p* < .05. Reliability measures were calculated in R using the *psych* function (version 2.0.12) based on Cronbach’s α (1951) and the average split half reliability (λ; see Guttman, 1945). Additionally, we calculated Cohen’s *d* effect sizes to estimate the magnitude of the difference between the C-RAT and LI-RAT accuracy, solution time, AHA! experience, and suddenness. To further visually compare the similarity between the LI-RAT and C-RAT, we provided Bland–Altman plots in Figure S7 in the Appendix for all measures (see Giavarina, 2015).

### Results and discussion

**Fig. 5:**
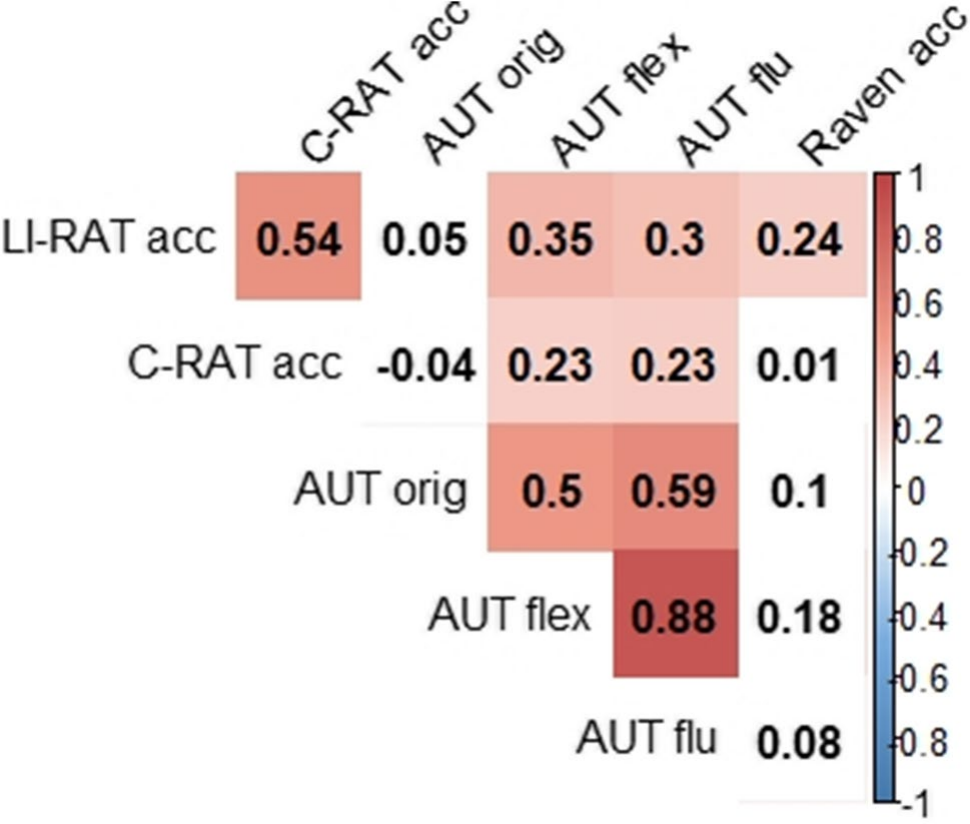
Correlation of LI-RAT accuracy with other creativity and problem-solving tasks across participants. *Note*. Acc = accuracy; C-RAT = compound remote associate task; Raven = Raven advanced progressive matrices; AUT = Alternative Uses Task; orig = originality; flex = flexibility; flu = fluency. The boxes in red represent significant correlations at a *p*-value level < .05, and the values are Spearman correlation coefficients.

As illustrated by Fig. 5, in general, LI-RAT accuracy was correlated with the two creativity tasks, C-RAT and AUT, as well as with Raven’s matrices (general problem-solving ability).

#### LI-RAT vs. C-RAT

Accuracy in the LI-RAT significantly correlated with accuracy in a different but language-based insight task, the C-RAT (*r*_*s*_ = .54, *p* < .05, *CI* = [.36–.71], Cohen’s *d* = 1.26). This relationship was still significant when controlling for a speed–accuracy tradeoff. The LI-RAT accuracy/solution time quotient correlated with the compound remote associate accuracy/solution time quotient significantly (*r*_*s*_ = .54, *p* < .05, *CI* = [.33–.72], Cohen’s *d* = 0.68). Response time for in both tasks also correlated significantly (*r*_*s*_ = .51, *p* < .05, *CI* = [.29–.68], Cohen’s *d* = 0.76). Additionally, there was a substantial relationship in the amount the AHA! experience (*r*_s_ = .72, *p* < .05, *CI* = [.56–.82], Cohen’s *d* = 0.14) and the amount of perceived suddenness of the solution (*r*_s_ = .59, *p* < .05, *CI* = [.41–.73], Cohen’s *d* = 0.23) in both tasks.

**Fig. 6:**
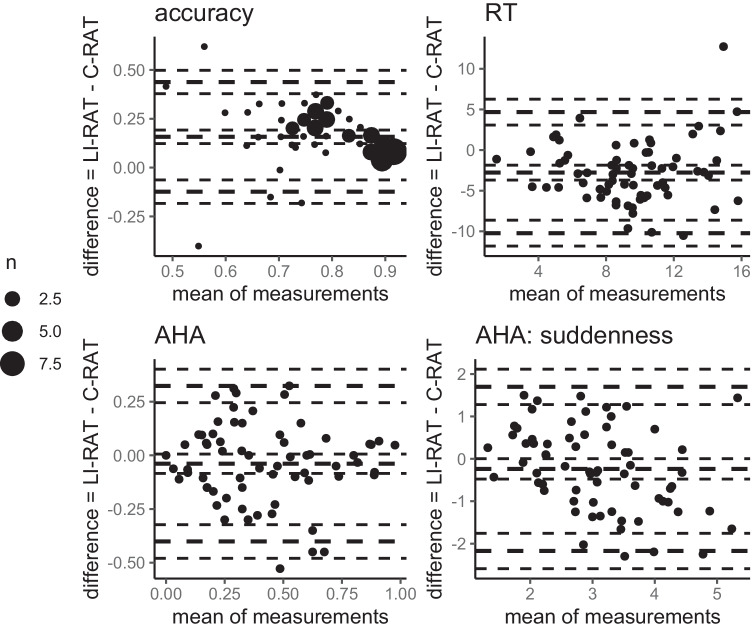
Bland–Altman plots for performance and AHA! experience measures comparing LI-RAT and C-RAT (subject level). *Note*. AHA = AHA! experience; RT = solution time. The outer thick lines represent the 1.96 standard deviation from the mean (middle thick line). The thinner lines around the thick lines represent the respective 95% confidence interval. The legend “*n*” indicating the amount of subjects per data point only refers to accuracy.

Note, accuracy was higher (*M* = .87, *SD* = 0.11), and solution time lower (*M* = 8.1, *SD* = 3.8) for the subset of 20 LI-RAT items compared to the subset of 20 C-RAT items (accuracy: *M* = .71, *SD* = .14; solution time: *M* = 10.8; *SD* = 3.5). This is also demonstrated in the shifted midline for accuracy and solution time of the Bland–Altman plots in Fig. 6 (upper left panel). However, this difference in task difficulty is due to the selection of 20 items in both insight problems.

#### LI-RAT vs. AUT

A moderate relationship between accuracy in the LI-RAT and two scores of the AUT was found: *flexibility* (*r*_*s*_ = .35, *p* < .05, *CI* = [.08–.55]) and *fluency* (*r*_*s*_ = .30, *p* < .05, *CI* = [.04–.53]). However, there was no evidence for a relationship between accuracy in the LI-RAT and the score *originality* of the AUT (*r*_*s*_ = .05, *CI* = [−.22–.30]).

#### LI-RAT vs Raven’s matrices

Finally, there was also evidence for a relationship between LI-RAT performance and Raven’s matrices, a nonverbal general problem-solving task. LI-RAT accuracy significantly correlated with accuracy in Raven’s matrices (*r*_*s*_ = .26, *p* < .05, *CI* = [.03–.47]). This relationship was still significant when dividing accuracy by solution time in both tasks: LI-RAT accuracy/solution time quotient significantly correlated with the Raven accuracy/solution time quotient (*r*_*s*_ = .42, *p* < .05, *CI* = [.16–.63]). Furthermore, response time between both tasks also correlated significantly (*r*_*s*_ = .33, *p* < .05, *CI* = [.08–.56]). Note, however, we did not find a significant correlation for accuracy between the C-RAT and the Raven matrices (*r*_s_ = .01, *p* > .05, *CI* = [−.23–.24]; see Fig. 5). This is in contrast to previous findings (Chuderski & Jastrzębski, 2018). The absence of this relationship could be due to the fact that we selected a certain subset of C-RAT items that differed from selections in previous studies (Chuderski & Jastrzębski, 2018). However, we did find a small correlation in solution time between C-RAT and Raven matrices (*r*_*s*_ = .27, *p* < .05, *CI* = [−.00–.51]).

Note, the tests were presented in a fixed order because we were interested in the correlation among tests. Randomizing the order would have introduced an additional source of error variance which could cause underestimation of the correlations. This is specifically the case when the tasks have *different* carry-over effects (Bell, 2013). For example, we assumed that the Raven task has a stronger fatigue effect due to strong working memory tasks compared to the AUT or LI-RAT, for example. However, due to the fixed order, we cannot exclude that a general increase in fatigue may have altered participant’s performance of those tasks executed towards the end of the battery like the AUT and the Raven tasks.

## General discussion

Most creativity tests are complex, which complicates the study of the critical cognitive components of creative problem-solving (Bowden & Jung-Beeman, 2003a). Therefore, simpler insight tasks, such as the RAT, have been developed and successfully applied to study creativity and different aspects of the insight process. Unfortunately, the RAT, like most creativity tests, is language-dependent, creating an obstacle for cross-cultural comparisons. Addressing this issue was our main motivation for developing a LI-RAT. In the current studies, we preliminarily normed the LI-RAT in English-, German-, and Spanish-speaking populations, measured the insight it generates, and compared it to popular creativity tests. The studies yielded two main findings. First, norming in the LI-RAT items with English-, German-, and Spanish-speaking populations confirmed that this test generates insight and that it can be used to compare different language samples. Second, comparisons with other creativity tests confirmed the validity of the LI-RAT as a creativity task. These two main findings are discussed in separate sections below.

### LI-RAT is comparable across language samples and elicits insight

Norming the items in English-, German-, and Spanish-speaking populations (study 1) showed that accuracy and RTs were comparable between all three samples, as the high between-item correlations demonstrate. Although a larger sample size per item (*n* = 100) could have provided more robust mean item values, this evidence provides support for our assumption that the LI-RAT is language-independent, in the sense that it can be used to compare different language populations. Note, however, that the LI-RAT items varied in their degree of difficulty (absolute values) between the samples. That is to say, while most items showed similar difficulty, a few strongly varied in difficulty across all three samples (maximum difference item difficulty difference = .5). This suggests that certain items are more susceptible to cultural differences than others. For this reason, choosing a subset of items with similar difficulty across samples is advisable when comparing insight problem-solving across different language samples. Importantly, we do not claim to have developed a *culture-free* but a *language-independent* creativity test. As noted in the Introduction, the availability of a LI-RAT is important for several reasons. First, the LI-RAT will facilitate cross-culture creativity studies even if the test itself may not be entirely culture-free. The reason for this is that the items of LI-RAT are identical in contrast to the translations of the RAT (e.g., Chermahini et al., 2012; Wu & Chen, 2017; Baba, 1982; Becker et al., 2020a) whose items are not identical. Using different items can introduce additional confounders when comparing across cultures (Behrens & Olteteanu, 2020). Second, the LI-RAT will facilitate the assessment of individuals with limited vocabulary, such as immigrants with partial knowledge of the language. Finally, the LI-RAT will facilitate investigating creativity processes in patients with language deficits due to brain lesions or other disorders.

From a cognitive neuroscience perspective, this last advantage is particularly noteworthy because the study of cognitive abilities in cognitively impaired patients is one of the main methods of cognitive neuroscience, the other being functional neuroimaging. Thus, a LI-RAT is necessary to investigate the neural bases of creativity in patients with language disorders. In fact, creativity is being actively studied in patients with frontotemporal dementia (for a review, see Palmiero et al., 2012) and Alzheimer’s disease (Cummings et al., 2008), both of which impair language at different states of the disorder.

In addition to demonstrating that the LI-RAT is comparable across populations in terms of performance parameters (accuracy and solution time), the results of study 1 showed that the LI-RAT generates insight. To be precise, LI-RAT items elicit an AHA! experience, and their solution is perceived with suddenness (one aspect of the AHA! experience) but to varying degrees. That is to say, LI-RAT items differ in their likelihood to elicit an AHA! experience (min. 16% – max. 96%). This is similar to the C-RAT, whose items can also be solved with or without an insight (Bowden & Jung-Beeman, 2003b). In fact, we found that insight in the LI-RAT correlates significantly across participants with insight in the C-RAT (study 2). Additionally, the individual LI-RAT items elicited a comparable AHA! experience between all three language samples as the between-item correlations demonstrate (study 1). However, the AHA! experience was the parameter that most strongly differed between the language samples in terms of absolute values. This is particularly interesting given that no substantial evidence for a difference in perceived suddenness upon solution between the language samples was found. Hence, it must be specifically the emotional response upon suddenly solving a LI-RAT item that differs between the samples. Prior studies have demonstrated that there are cultural differences in emotional responses and how they are experienced and evaluated by the individual (Lim, 2016; Lewis et al., 2010; Senft et al., 2020). Because there is currently no systematic investigation of cross-cultural differences in the AHA! experience, future studies should further investigate this matter using for example the LI-RAT including more diverse language samples. This becomes particularly relevant as different aspects of the AHA! experience (emotional response vs. suddenness) seem to be affected differently by cultural differences.

### The LI-RAT correlates with popular creativity and general problem-solving tasks

Study 2 compared the LI-RAT to two popular creativity tasks, the Compound Remote Associate Task (C-RAT) and the Alternative Uses Task (AUT). We found that the LI-RAT significantly correlated with the language-dependent C-RAT. This was expected given that both the LI-RAT and the C-RAT involve finding remote associations and that the tasks were matched according to number of items, task difficulty, and task procedure. However, there was still a substantial amount of variance left unexplained between both tasks (*r* = .46). This could reflect the fact that the LI-RAT incorporates a visual component and requires thinking of remote associations not only in the conceptual but also in the perceptual domain.

In addition to its relationship with the C-RAT, the LI-RAT shares variance with two sub-scores of the AUT—another widely used creativity task. LI-RAT accuracy was positively correlated with *fluency* and *flexibility* but not with *originality* of the AUT. Similarly, the C-RAT correlated with *flexibility* and *fluency* but not with *originality* of the AUT. Salvi et al. (2020), who validated an Italian verbal version of the RAT, also found a correlation between *fluency* and *flexibility* but not with *originality* of the AUT. The consistent relationship between the LI-RAT/C-RAT and AUT fluency and flexibility could be explained by an overall ability to fluently produce possible solutions given a set of constraints (in the case of the LI-RAT, finding a solution given two perceptually/conceptually related pictures, and in the case of the AUT, producing many possible unusual uses given an object). This is also consistent with the positive relationship between the LI-RAT and verbal semantic fluency which also requires a fluent production of words given a certain category. In contrast, the reason for the lack of correlation between the *originality* sub-score and the LI-RAT/C-RAT could be due to the low reliability of the *originality* score. Additionally, the lack of correlation could be due to the fact that the need for original responses is less emphasized in the LI-RAT/RAT than in the AUT.

One factor that could account for creativity components that the LI-RAT shares with C-RAT and AUT is the ability to think beyond short-distance semantic associations into longer relationships in the semantic memory network. This relates well to Mednick’s creativity model of associative hierarchies (Mednick, 1962). The model assumes that more creative individuals have flatter associative hierarchies compared to less creative ones and, as a consequence, can more fluently retrieve remote associative elements, which can be combined to form creative ideas.

The necessity to retrieve more or less remote associative elements can also be investigated by comparing the relational properties between the problem elements (cues and target) and between the items. Prior research investigating conceptual similarities in verbal C-RAT items via semantic distances measures has been successful in disentangling the different sources of task difficulty contributing to insight (Becker et al., 2020a). Similarly, the idea generation process in the AUT has also been decomposed using semantic distance measures (e.g., Hass, 2017). Hence, one promising future endeavor would be to investigate the component processes of insight based on the relational properties (perceptual and conceptual similarities) between all three problem elements in the LI-RAT.

Apart from its relationship to other creativity measures, LI-RAT accuracy shares significant variance with accuracy in more general cognitive-control tasks measuring verbal fluency and language-independent problem-solving ability. The small but significant correlation between the Raven matrices and LI-RAT accuracy of *r* = .24 is consistent with the correlation that Chuderski and Jastrzębski (2018) found between the Raven matrices and the (verbal) RAT (*r* = .28). Both results are in line with dual-process models assuming that creativity does not only depend on associative processes but rather arises as a result of an interaction between associative *and* control processes (Beaty et al., 2014, 2016; Benedek et al., 2012; note, however, the missing evidence for a relationship in accuracy between the C-RAT and Raven’s matrices in study 2).

### Comparison of the LI-RAT to a visual RAT

Apart from the existing and validated language-independent tests (TTCT, TCIA) already mentioned above, a promising set of visual RAT items has been recently created including three pictures that need to be related to each other instead of three words (Olteteanu & Zunjani, 2020). This visual RAT and the LI-RAT are similar in the sense that pictures are presented and that the solver is required to find a solution that is related to all presented cues/pictures. However, there are four important differences. First, neither the individual items of the visual RAT nor its normative data are freely available. The usefulness of a new test critically depends on the availability of this information. Second, the amount of different items is currently limited in the visual RAT (*n* = 46), making it less suitable for neurocognitive methods than the LI-RAT (*n* = 121). For example, both event-related potentials (ERPs) and event-related fMRI studies require dozens of items per condition, so most of these studies use close to over 100 items. Third, the LI-RAT requires only two (instead of three) pictures to solve the problem and is therefore even simpler in its presentation than the visual RAT. This can be particularly useful when investigating insight using neurocognitive methods such as fMRI, EEG, or eye-tracking methods because of better experimental controllability of the stimulus material. Furthermore, the relationships (e.g., semantic, visual) between the problem elements and between the problem elements and the solution are likely nonlinear, and hence, the more problem elements there are, the more difficult it becomes to disentangle those individual relationships experimentally. Fourth, the visual RAT is only visual in terms of translating the picture cues into meaningful concepts. Hence, once the cues are encoded by the solver, the search for the solution is mostly conceptual because the relationship between the cues is only conceptual—similar to the RAT. In contrast, the cues in the LI-RAT are not related conceptually, and finding the solution requires a conceptual *and* a visual search in parallel. Therefore, the two tasks are quite different and can be used for different research goals, making them complementary.

## Conclusion

In summary, 121 LI-RAT stimuli have been presented and preliminarily validated as part of this paper. The items presented here are language-independent, simple, and physically compact. They are therefore suited to study different aspects of insight and creativity over different language populations. By providing item-specific information on performance and AHA! experience in addition to conceptual and perceptual similarity measures, we hope to encourage the further use of those LI-RAT items for future research.

## Supplementary Information

ESM 1 (DOCX 161 kb)

## Footnotes

1 The C-RAT is almost identical to the RAT with the difference that the cue words in the C-RAT are syntactically related to the solution via compound words; see Bowden & Jung-Beeman, 2003a, b.

2 We decided for a cutoff criterion of at least 35% item difficulty because the items are supposed to be suitable for functional magnetic resonance imaging (fMRI) experiments. As the signal-to-noise ratio is low for fMRI, we wanted to ensure that participants could solve sufficient LI-RAT problems on average to increase this ratio.

3 Note, no prime was presented for this study (see Becker et al., 2020a).
