# Supplementary material for: Assessing creativity independently of language: A language-independent remote associate task (LI-RAT)
Source: Behav Res Methods. 2022 Mar 10;55(1):85–102. doi: 10.3758/s13428-021-01773-5 (PMC9918581; doi:10.3758/s13428-021-01773-5)

Appendix

**Table S1**

*Normative data on 121 LI-RAT stimuli – English sample*

|  |  |  |  |  |  |  | English sample | | | | | | | |
| --- | --- | --- | --- | --- | --- | --- | --- | --- | --- | --- | --- | --- | --- | --- |
| LI-RAT perceptual cue | LI-RAT conceptual cue | LI-RAT solution | conceptual similarity (conc. cue-  solution | conceptual similarity (perc. cue- solution | perceptual similarity (conc. cue-  solution | perceptual similarity (perc. cue-  solution | button press in % | Accu-  racy in % | RT (sec) | RT if correct (sec) | AHA: Suddenness (1 - 7) | AHA in % | N | N with altern. sol. |
| trolley | ice crystal | sleigh | 0,31 | 0,30 | 0,42 | 0,13 | 0,94 | 0,58 | 7,77 | 4,43 | 3,15 | 0,48 | 64 | 0 |
| bag | key | lock | 0,33 | 0,34 | 0,57 | 0,19 | 0,90 | 0,42 | 12,68 | 10,92 | 4,21 | 0,63 | 78 | 0 |
| bat | garlic | vampire | 0,51 | 0,32 | 0,13 | 0,03 | 0,85 | 0,64 | 11,49 | 8,70 | 3,26 | 0,39 | 82 | 0 |
| beer glass | podium | trophy | 0,51 | 0,46 | 0,37 | 0,11 | 0,92 | 0,48 | 9,96 | 9,50 | 3,86 | 0,49 | 91 | 1 |
| cauliflower | cine camera | popcorn | 0,64 | 0,48 | 0,47 | 0,01 | 0,85 | 0,62 | 9,34 | 4,98 | 3,08 | 0,43 | 87 | 0 |
| bomb | firtree | christmas bauble | 0,40 | 0,57 | 0,42 | 0,09 | 0,99 | 0,78 | 7,02 | 5,48 | 2,63 | 0,37 | 88 | 0 |
| boomerang | strawberry | banana | 0,37 | 0,71 | 0,49 | 0,14 | 0,99 | 0,94 | 4,40 | 3,91 | 2,14 | 0,32 | 80 | 0 |
| bowling ball | palmtree | coconut | 0,49 | 0,40 | 0,26 | 0,04 | 0,93 | 0,83 | 5,16 | 4,28 | 1,86 | 0,26 | 76 | 0 |
| bowling pins | wine glass | bottle | 0,33 | 0,70 | 0,12 | 0,31 | 0,91 | 0,78 | 11,34 | 9,95 | 3,69 | 0,54 | 80 | 10 |
| bowtie | bug | butterfly | 0,40 | 0,54 | 0,11 | 0,37 | 0,95 | 0,68 | 8,70 | 7,87 | 3,76 | 0,57 | 22 | 0 |
| boxing | sushi | lobster | 0,39 | 0,47 | 0,38 | 0,25 | 0,87 | 0,45 | 13,35 | 9,81 | 3,82 | 0,54 | 83 | 0 |
| branch | deer | antlers | 0,50 | 0,42 | 0,37 | 0,21 | 0,96 | 0,86 | 5,19 | 4,77 | 2,00 | 0,32 | 74 | 0 |
| bridge | weather | rainbow | 0,39 | 0,36 | 0,2 | 0,2 | 0,97 | 0,60 | 9,37 | 7,44 | 3,19 | 0,43 | 78 | 2 |
| broccoli | radioactive | atomic bomb | 0,35 | 0,16 | 0,23 | -0,02 | 0,95 | 0,62 | 9,67 | 7,58 | 3,51 | 0,51 | 93 | 0 |
| ironing board | wetsuit | surfboard | 0,55 | 0,58 | 0,19 | 0,25 | 0,99 | 0,86 | 5,35 | 4,35 | 2,33 | 0,35 | 83 | 0 |
| brush | lipstick | mascara | 0,44 | 0,52 | 0,53 | 0,43 | 0,97 | 0,75 | 8,53 | 7,39 | 3,07 | 0,36 | 71 | 3 |
| flatiron | salmon | ship | 0,39 | 0,31 | 0,46 | 0,23 | 0,88 | 0,48 | 10,77 | 9,94 | 4,29 | 0,63 | 90 | 0 |
| bullet | satellite | rocket | 0,54 | 0,44 | 0,32 | 0,18 | 0,97 | 0,81 | 7,50 | 6,79 | 3,31 | 0,46 | 86 | 0 |
| can | plunger | tiolet | 0,34 | 0,37 | 0,24 | 0,19 | 0,99 | 0,87 | 6,50 | 5,56 | 2,76 | 0,33 | 69 | 0 |
| cat | pyramid | sphinx | 0,44 | 0,46 | 0,17 | 0,2 | 0,95 | 0,81 | 6,77 | 6,13 | 2,33 | 0,25 | 83 | 0 |
| ceiling fan | conch | starfish | 0,51 | 0,43 | 0,19 | 0,27 | 0,91 | 0,65 | 9,69 | 7,62 | 3,55 | 0,44 | 86 | 2 |
| celery | swing | slide | 0,23 | 0,20 | 0,31 | 0,25 | 0,96 | 0,77 | 7,56 | 6,02 | 2,97 | 0,32 | 75 | 0 |
| cigarette | chalkboard | chalk | 0,30 | 0,34 | 0,31 | 0,12 | 1,00 | 0,91 | 4,29 | 3,43 | 1,99 | 0,30 | 68 | 0 |
| corset | stopwatch | hourglass | 0,32 | 0,54 | 0,13 | 0,08 | 0,90 | 0,63 | 10,02 | 7,49 | 3,13 | 0,40 | 79 | 0 |
| dalmatian | milk | cow | 0,51 | 0,47 | 0,29 | 0,06 | 0,91 | 0,60 | 9,99 | 7,84 | 3,18 | 0,44 | 85 | 0 |
| triangle | notes | triangle | 0,39 | 0,34 | 0,59 | 0,22 | 0,94 | 0,75 | 11,16 | 10,04 | 3,61 | 0,60 | 77 | 0 |
| ferriswheel | wheat | water mill | 0,47 | 0,34 | 0,12 | 0,05 | 0,92 | 0,45 | 12,42 | 10,69 | 4,10 | 0,56 | 76 | 5 |
| flower | vinyl | phonograph | 0,38 | 0,54 | 0,04 | 0,09 | 0,96 | 0,75 | 8,40 | 8,65 | 3,34 | 0,49 | 77 | 10 |
| football | patch | scar | 0,26 | 0,40 | 0,35 | 0,2 | 0,86 | 0,35 | 16,56 | 12,50 | 4,46 | 0,66 | 82 | 5 |
| filmcan | circuit | battery | 0,41 | 0,45 | 0,43 | 0,08 | 0,86 | 0,39 | 11,72 | 9,08 | 4,63 | 0,73 | 85 | 0 |
| friedegg | tape | CD | 0,39 | 0,73 | 0,39 | 0,14 | 0,94 | 0,71 | 9,63 | 7,83 | 3,54 | 0,53 | 80 | 16 |
| gasbottle | fire | fire extinguisher | 0,58 | 0,37 | 0,46 | 0,16 | 1,00 | 0,67 | 7,74 | 5,57 | 3,15 | 0,60 | 72 | 0 |
| watering can | giraffe | elephant | 0,41 | 0,69 | 0,06 | 0,13 | 0,88 | 0,55 | 11,10 | 7,44 | 3,58 | 0,52 | 76 | 1 |
| bell | scarf | hat | 0,36 | 0,38 | 0,25 | 0,29 | 0,90 | 0,60 | 15,09 | 14,88 | 4,58 | 0,65 | 72 | 1 |
| golf ball | astronaut | moon | 0,40 | 0,29 | 0,37 | 0,04 | 0,99 | 0,75 | 6,91 | 6,37 | 2,56 | 0,39 | 72 | 0 |
| grapes | party hat | balloons | 0,38 | 0,53 | 0,37 | 0,21 | 0,94 | 0,81 | 6,60 | 5,56 | 2,47 | 0,25 | 77 | 0 |
| gun | comb | hairdryer | 0,42 | 0,39 | 0,47 | 0,22 | 0,96 | 0,78 | 11,15 | 8,79 | 3,21 | 0,28 | 89 | 0 |
| handcuff | eye | glasses | 0,38 | 0,37 | 0,35 | 0,19 | 0,99 | 0,85 | 5,50 | 4,80 | 2,34 | 0,29 | 74 | 0 |
| phone | sliderule | calculator | 0,62 | 0,58 | 0,38 | 0,32 | 0,96 | 0,90 | 6,51 | 5,98 | 2,42 | 0,29 | 82 | 0 |
| hedgehog | barrette | brush | 0,35 | 0,59 | 0,22 | 0,3 | 0,94 | 0,56 | 7,74 | 6,21 | 3,86 | 0,62 | 81 | 2 |
| ice cone | head-phones | microphone | 0,42 | 0,60 | 0,24 | 0,26 | 0,98 | 0,74 | 6,89 | 4,93 | 2,98 | 0,33 | 90 | 0 |
| igloo | baseball | baseball cap | 0,39 | 0,74 | 0,4 | 0,1 | 0,92 | 0,36 | 11,50 | 9,83 | 4,14 | 0,59 | 83 | 1 |
| insect | car | helicopter | 0,39 | 0,54 | 0,48 | 0,21 | 0,99 | 0,75 | 7,02 | 6,21 | 3,13 | 0,44 | 82 | 0 |
| cable reel | filmtimer | film reel | 0,47 | 0,43 | 0,3 | 0,17 | 0,97 | 0,79 | 9,04 | 8,69 | 3,05 | 0,51 | 75 | 0 |
| necklace | dog | collar | 0,48 | 0,34 | 0,1 | 0,02 | 0,99 | 0,92 | 5,02 | 4,76 | 2,29 | 0,36 | 91 | 0 |
| button | hole | gully | 0,38 | 0,46 | 0,2 | 0,22 | 0,87 | 0,23 | 13,38 | 9,59 | 4,40 | 0,63 | 83 | 1 |
| tin | baby | crib | 0,37 | 0,30 | 0,15 | 0,16 | 0,99 | 0,88 | 7,67 | 6,41 | 3,01 | 0,46 | 73 | 0 |
| crab | nail1 | pliers | 0,33 | 0,28 | 0,24 | 0,2 | 0,83 | 0,41 | 16,09 | 14,44 | 4,68 | 0,68 | 88 | 0 |
| ladder | loco | railtrack | 0,29 | 0,42 | 0,25 | 0,12 | 0,97 | 0,72 | 11,25 | 10,85 | 3,24 | 0,41 | 61 | 0 |
| dragonfly | doll | fairy | 0,42 | 0,34 | 0,22 | 0,45 | 0,85 | 0,53 | 12,45 | 10,31 | 4,03 | 0,61 | 85 | 2 |
| linden leaf | card game | spade | 0,30 | 0,27 | 0,21 | 0,15 | 0,93 | 0,64 | 9,29 | 7,09 | 3,01 | 0,44 | 84 | 11 |
| ruler | pool | diving board | 0,35 | 0,31 | 0,34 | 0,47 | 0,92 | 0,56 | 9,15 | 6,09 | 3,56 | 0,48 | 84 | 1 |
| magnifier | pot | pan | 0,41 | 0,64 | 0,52 | 0,35 | 0,99 | 0,67 | 7,50 | 6,34 | 2,96 | 0,56 | 79 | 1 |
| magnet | shoes | horseshoe | 0,36 | 0,42 | 0,52 | 0,3 | 0,92 | 0,56 | 9,91 | 7,70 | 3,77 | 0,59 | 80 | 0 |
| spiky ball | fish | blowfish | 0,55 | 0,55 | 0,15 | 0,25 | 0,99 | 0,60 | 9,20 | 8,10 | 3,45 | 0,54 | 77 | 20 |
| tape rule | frog | snail | 0,41 | 0,64 | 0,21 | 0,1 | 0,94 | 0,55 | 9,59 | 8,54 | 3,56 | 0,54 | 88 | 0 |
| microwave | oscar | TV | 0,57 | 0,31 | 0,52 | 0,05 | 0,91 | 0,42 | 12,91 | 9,93 | 4,51 | 0,63 | 78 | 1 |
| mosaic | poster | puzzle | 0,63 | 0,39 | 0,18 | 0,04 | 0,77 | 0,08 | 14,24 | 19,42 | 4,93 | 0,82 | 80 | 0 |
| marble | trampoline | rubber ball | 0,51 | 0,50 | 0,3 | 0,09 | 0,83 | 0,64 | 14,52 | 13,59 | 4,49 | 0,65 | 69 | 1 |
| screwnut | bee | honeycomb | 0,53 | 0,45 | 0,17 | 0,1 | 0,95 | 0,72 | 9,48 | 7,65 | 3,41 | 0,51 | 80 | 0 |
| slug | coffee | croissant | 0,43 | 0,51 | 0,28 | 0,27 | 0,82 | 0,35 | 14,74 | 9,03 | 4,67 | 0,68 | 77 | 2 |
| hook | suction cup | octopus | 0,50 | 0,54 | 0,32 | 0,13 | 0,88 | 0,40 | 12,43 | 9,48 | 3,87 | 0,61 | 80 | 0 |
| folder | clef | accordion | 0,36 | 0,33 | 0,37 | 0,09 | 0,98 | 0,76 | 7,31 | 5,71 | 2,87 | 0,51 | 87 | 0 |
| paper pile | coin | banknote | 0,35 | 0,54 | 0,13 | 0,09 | 0,96 | 0,86 | 8,86 | 8,61 | 3,51 | 0,60 | 79 | 10 |
| pear | candle | light bulb | 0,60 | 0,54 | 0,24 | 0,25 | 0,83 | 0,36 | 13,10 | 9,66 | 4,33 | 0,56 | 80 | 0 |
| pen | drums | drumsticks | 0,49 | 0,43 | 0,53 | 0,16 | 1,00 | 0,65 | 5,30 | 4,55 | 2,39 | 0,48 | 76 | 0 |
| penguin | cross | nun | 0,44 | 0,36 | 0,26 | 0,17 | 0,79 | 0,37 | 12,46 | 7,73 | 4,31 | 0,58 | 79 | 1 |
| peacock | ventilator | fan | 0,38 | 0,36 | 0,31 | 0,17 | 0,99 | 0,89 | 10,68 | 9,97 | 3,33 | 0,61 | 93 | 0 |
| mushroom | cagoule | umbrella | 0,32 | 0,59 | 0,3 | 0,23 | 0,96 | 0,61 | 9,28 | 7,53 | 3,68 | 0,57 | 72 | 1 |
| pistachio | pearls | clam | 0,62 | 0,53 | 0,21 | 0,25 | 0,92 | 0,59 | 10,64 | 8,20 | 3,64 | 0,56 | 80 | 9 |
| pocket watch | world map | compass | 0,32 | 0,41 | 0,48 | 0,2 | 0,91 | 0,51 | 13,14 | 11,12 | 3,79 | 0,48 | 85 | 0 |
| polaroid | mailbox | stamp | 0,45 | 0,47 | 0,27 | 0,07 | 0,92 | 0,52 | 9,28 | 4,46 | 3,51 | 0,61 | 91 | 39 |
| porcupine | dustpan | broom | 0,39 | 0,58 | 0,13 | 0,29 | 0,98 | 0,77 | 6,72 | 5,84 | 2,83 | 0,41 | 84 | 0 |
| pumpkin | trainers | basketball | 0,35 | 0,41 | 0,35 | 0,13 | 0,90 | 0,73 | 6,58 | 5,27 | 2,62 | 0,35 | 77 | 0 |
| tire | hand | ring | 0,32 | 0,27 | 0,25 | 0,22 | 0,95 | 0,68 | 10,38 | 8,73 | 3,37 | 0,42 | 77 | 2 |
| cleanser | bird | swan* | 0,17 | 0,66 | 0,14 | 0,18 | 0,95 | 0,82 | 6,92 | 5,49 | 2,64 | 0,39 | 79 | 32 |
| lifesaver | muffin | doughnut | 0,44 | 0,61 | 0,18 | 0,28 | 0,99 | 0,90 | 4,49 | 3,69 | 2,18 | 0,38 | 74 | 2 |
| pipe system | mouse | maze | 0,39 | 0,32 | 0,33 | 0 | 0,95 | 0,79 | 7,15 | 5,37 | 3,13 | 0,48 | 87 | 0 |
| roots | blood | veins | 0,34 | 0,38 | 0,2 | 0,12 | 0,99 | 0,89 | 6,34 | 5,82 | 2,81 | 0,39 | 81 | 0 |
| salt shaker | racket | shuttlecock | 0,37 | 0,36 | 0,42 | 0,14 | 0,80 | 0,50 | 12,58 | 10,17 | 3,97 | 0,49 | 76 | 0 |
| box | motherboard | laptop | 0,51 | 0,61 | 0,19 | 0,26 | 0,96 | 0,79 | 6,26 | 5,20 | 2,88 | 0,34 | 68 | 1 |
| hose | poison | snake | 0,31 | 0,29 | 0,07 | 0,22 | 0,98 | 0,76 | 8,54 | 6,97 | 3,05 | 0,37 | 83 | 0 |
| sponge1 | cow | cheese | 0,41 | 0,44 | 0,24 | 0,1 | 0,95 | 0,79 | 6,45 | 4,70 | 2,64 | 0,37 | 88 | 0 |
| sponge2 | sharpener | eraser | 0,51 | 0,71 | 0,45 | 0,16 | 0,93 | 0,66 | 9,34 | 7,91 | 3,60 | 0,49 | 73 | 0 |
| soap | monitor | computer mouse | 0,44 | 0,53 | 0,56 | 0,17 | 0,91 | 0,50 | 9,08 | 6,07 | 3,07 | 0,42 | 80 | 0 |
| ski | noodles | chopsticks | 0,48 | 0,56 | 0,24 | 0,01 | 1,00 | 0,86 | 6,38 | 5,06 | 2,73 | 0,38 | 80 | 0 |
| sleeping bag | egypt | mummy* | 0,32 | 0,45 | 0,39 | 0,1 | 1,00 | 0,88 | 5,90 | 4,81 | 2,51 | 0,32 | 75 | 20 |
| snake | pants | belt | 0,27 | 0,39 | 0,42 | -0,02 | 0,96 | 0,75 | 7,61 | 5,85 | 2,91 | 0,40 | 85 | 0 |
| spade | cake | cake server | 0,57 | 0,41 | 0,45 | 0,03 | 0,90 | 0,54 | 10,91 | 11,75 | 4,04 | 0,67 | 83 | 0 |
| coffee table | fly | spider | 0,25 | 0,50 | 0,39 | 0,31 | 0,95 | 0,64 | 12,25 | 10,17 | 4,40 | 0,73 | 88 | 0 |
| noodle | bottle opener | corkscrew | 0,45 | 0,69 | 0,24 | 0,47 | 0,95 | 0,72 | 10,05 | 8,65 | 2,90 | 0,38 | 82 | 0 |
| star fruit | saturn | star | 0,32 | 0,50 | 0,31 | 0,22 | 0,96 | 0,60 | 11,62 | 8,61 | 3,75 | 0,63 | 70 | 9 |
| socket | face mask | face | 0,33 | 0,41 | 0,12 | 0,08 | 0,97 | 0,57 | 10,56 | 7,69 | 3,79 | 0,57 | 74 | 2 |
| pin | candy | lollipop | 0,39 | 0,47 | 0,73 | 0,25 | 0,99 | 0,85 | 6,61 | 5,89 | 2,72 | 0,42 | 82 | 0 |
| stamp | nail2 | hammer | 0,37 | 0,38 | 0,33 | 0,53 | 0,95 | 0,77 | 9,31 | 8,72 | 3,96 | 0,70 | 95 | 0 |
| stone | stethos-cope | heart | 0,35 | 0,39 | 0,2 | 0,2 | 1,00 | 0,95 | 3,24 | 3,20 | 1,80 | 0,30 | 83 | 0 |
| street light | crown | scepter | 0,28 | 0,40 | 0,53 | 0,16 | 0,92 | 0,52 | 9,88 | 7,04 | 3,55 | 0,55 | 72 | 0 |
| match | burger | fries | 0,44 | 0,58 | 0,35 | 0,28 | 0,97 | 0,71 | 7,07 | 5,37 | 2,77 | 0,44 | 74 | 1 |
| T | sweatpants | t-shirt | 0,25 | 0,60 | 0,45 | 0,38 | 0,94 | 0,80 | 10,33 | 9,56 | 3,83 | 0,61 | 80 | 4 |
| lamp | blanket | tent | 0,45 | 0,56 | 0,22 | 0,25 | 0,99 | 0,85 | 4,63 | 4,12 | 2,57 | 0,35 | 78 | 0 |
| tiefighter | weight bench | barbell | 0,40 | 0,58 | 0,37 | 0,19 | 0,97 | 0,85 | 7,49 | 6,95 | 3,11 | 0,38 | 75 | 0 |
| whiteout | mousetrap | mouse | 0,45 | 0,39 | 0,32 | 0,1 | 0,91 | 0,63 | 11,93 | 9,61 | 4,19 | 0,68 | 77 | 0 |
| paddle | crosswalk | stop sign | 0,48 | 0,51 | 0,24 | 0,32 | 0,98 | 0,86 | 6,52 | 6,01 | 2,62 | 0,42 | 86 | 0 |
| evil eye perl | binoculars | eye* | 0,33 | 0,38 | 0,24 | 0,18 | 0,93 | 0,75 | 7,06 | 5,68 | 3,15 | 0,48 | 71 | 10 |
| tower | flashlight | light house | 0,51 | 0,31 | 0,21 | 0,08 | 0,97 | 0,38 | 10,22 | 9,04 | 3,86 | 0,52 | 79 | 0 |
| tv tower | pills | syringe | 0,17 | 0,54 | 0,25 | 0,4 | 0,96 | 0,81 | 6,71 | 5,43 | 3,12 | 0,49 | 77 | 35 |
| tyrannosaur | baby sling | kangaroo | 0,49 | 0,45 | 0,31 | 0,13 | 0,84 | 0,41 | 15,32 | 12,32 | 4,67 | 0,69 | 76 | 0 |
| ufo | hockey helmet | puck | 0,33 | 0,50 | 0,31 | 0,13 | 0,93 | 0,55 | 9,60 | 8,85 | 3,43 | 0,51 | 74 | 0 |
| spatula | mosquito | fly swatter | 0,53 | 0,37 | 0,36 | 0,28 | 0,96 | 0,87 | 5,00 | 4,50 | 2,18 | 0,34 | 85 | 0 |
| vase | coffin | urn | 0,43 | 0,67 | 0,19 | 0,08 | 0,99 | 0,82 | 8,41 | 6,69 | 2,89 | 0,39 | 77 | 0 |
| power strip | turn sign | traffic lights | 0,31 | 0,48 | 0,24 | 0,14 | 0,93 | 0,70 | 9,12 | 7,05 | 3,45 | 0,44 | 85 | 0 |
| birdcage | gavel | prison | 0,38 | 0,17 | 0,25 | 0,07 | 0,95 | 0,74 | 9,29 | 6,85 | 3,06 | 0,40 | 82 | 0 |
| scale | playground | seesaw | 0,45 | 0,58 | 0,28 | 0,18 | 0,98 | 0,74 | 6,98 | 5,58 | 2,72 | 0,38 | 82 | 0 |
| walnut | skeleton | brain | 0,37 | 0,47 | 0,33 | 0,08 | 1,00 | 0,72 | 7,05 | 4,91 | 2,52 | 0,29 | 82 | 18 |
| helmet | chameleon | turtle | 0,30 | 0,43 | 0,47 | 0,39 | 0,94 | 0,53 | 8,06 | 6,15 | 2,94 | 0,44 | 67 | 0 |
| washer | tripod | camera | 0,56 | 0,69 | 0,45 | 0 | 0,97 | 0,79 | 6,20 | 5,52 | 2,71 | 0,46 | 68 | 0 |
| batting | sugar shaker | cotton candy | 0,51 | 0,46 | 0,27 | 0,3 | 0,96 | 0,10 | 10,81 | 12,62 | 3,81 | 0,54 | 73 | 0 |
| wheel | oven | pizza | 0,32 | 0,38 | 0,09 | 0,21 | 0,92 | 0,63 | 9,61 | 7,47 | 3,38 | 0,49 | 75 | 0 |
| zebra | road | crosswalk | 0,29 | 0,28 | 0,09 | 0,22 | 0,87 | 0,29 | 13,74 | 9,66 | 4,29 | 0,67 | 86 | 0 |
| sugar cane | wheelchair | cane | 0,34 | 0,54 | 0,16 | 0,13 | 0,98 | 0,86 | 6,32 | 5,44 | 3,09 | 0,43 | 83 | 0 |
| onion dome | carrot | onion* | 0,44 | 0,72 | 0,12 | 0,22 | 0,95 | 0,74 | 8,94 | 8,67 | 3,63 | 0,47 | 74 | 24 |
| *Note*. Button press = correctly and incorrectly solved items; RT = solution time (in seconds); RT if correct = solution time when solution was correct (in seconds); AHA = AHA! experience in %; AHA: Suddenness = Suddenness of the solution (part of AHA! experience) on a scale from 0 (continuous) to 6 (very sudden); N = amount of participants; Alternative Solutions = sum of correctly named alternative solutions; N with altern. sol. = amount of subjects with correct alternative solution; conc. cue-solution = similarity between conceptual cue and the solution; perc. cue-solution = similarity between perceptual cue to the solution. The asterisk in the LI-RAT solution column represents items that had 3 or 4 correct alternative solutions in the English sample. The correspondent pictures to the cues are freely available online here: https://github.com/MaxiBecker/LI-RAT.git. | | | | | | | | | | | | | | |

**Table S2**

*Normative data on 121 LI-RAT stimuli – German and Spanish sample*

|  |  |  | **German sample** | | | | | | | | **Spanish sample** | | | | | | | |
| --- | --- | --- | --- | --- | --- | --- | --- | --- | --- | --- | --- | --- | --- | --- | --- | --- | --- | --- |
| LI-RAT perceptual cue | LI-RAT conceptual cue | LI-RAT solution | button press in % | accuracy in % | RT (sec) | RT if correct (sec) | AHA: Suddenness (1 - 7) | AHA in % | N | N with altern. sol. | button press in % | accuracy in % | RT (sec) | RT if correct (sec) | AHA: Suddenness (1 - 7) | AHA in % | N | N with altern. sol. |
| trolley | ice crystal | sleigh | 0,89 | 0,54 | 10,24 | 4,62 | 3,56 | 0,52 | 28 | 0 | 0,97 | 0,53 | 11,35 | 8,87 | 3,49 | 0,40 | 36 | 0 |
| bag | key | lock | 0,80 | 0,30 | 14,94 | 8,93 | 4,67 | 0,58 | 30 | 0 | 0,92 | 0,33 | 14,29 | 9,90 | 4,08 | 0,50 | 39 | 0 |
| bat | garlic | vampire | 0,82 | 0,68 | 5,36 | 4,14 | 2,39 | 0,28 | 22 | 0 | 0,86 | 0,69 | 11,55 | 9,97 | 2,88 | 0,36 | 29 | 0 |
| beer glass | podium | trophy‡ | 0,96 | 0,72 | 9,47 | 7,42 | 2,96 | 0,38 | 25 | 0 | 0,97 | 0,75 | 10,71 | 9,08 | 2,97 | 0,26 | 32 | 0 |
| cauliflower | cine camera | popcorn | 0,78 | 0,44 | 12,32 | 6,71 | 3,36 | 0,40 | 32 | 0 | 0,96 | 0,73 | 7,67 | 5,62 | 2,68 | 0,24 | 26 | 0 |
| bomb | firtree | christmas bauble | 0,96 | 0,86 | 7,46 | 4,75 | 3,11 | 0,26 | 28 | 0 | 1,00 | 0,76 | 6,39 | 5,12 | 2,50 | 0,29 | 34 | 0 |
| boomerang | strawberry | banana | 1,00 | 0,91 | 3,67 | 3,27 | 2,32 | 0,32 | 22 | 0 | 0,96 | 0,91 | 6,79 | 6,06 | 2,68 | 0,05 | 23 | 0 |
| bowling ball | palmtree | coconut | 1,00 | 0,93 | 5,20 | 4,51 | 2,04 | 0,18 | 28 | 0 | 1,00 | 0,85 | 3,81 | 2,66 | 2,33 | 0,19 | 27 | 1 |
| bowling pins | wine glass | bottle*# | 0,97 | 0,72 | 12,24 | 9,87 | 3,94 | 0,63 | 36 | 5 | 1,00 | 0,78 | 10,05 | 8,32 | 2,94 | 0,22 | 32 | 4 |
| bowtie | bug | butterfly | 0,89 | 0,52 | 11,66 | 7,63 | 3,83 | 0,33 | 27 | 0 | 0,88 | 0,53 | 11,72 | 8,62 | 3,27 | 0,43 | 34 | 2 |
| boxing | sushi | lobster | 0,74 | 0,39 | 12,65 | 6,63 | 4,17 | 0,70 | 31 | 0 | 0,94 | 0,44 | 15,25 | 13,75 | 4,50 | 0,53 | 36 | 1 |
| branch | deer | antlers | 1,00 | 0,89 | 6,52 | 6,95 | 2,29 | 0,32 | 28 | 0 | 1,00 | 0,92 | 6,19 | 5,95 | 2,31 | 0,18 | 39 | 1 |
| bridge | weather | rainbow | 0,94 | 0,77 | 7,36 | 5,94 | 3,03 | 0,31 | 31 | 0 | 1,00 | 0,58 | 12,74 | 9,78 | 3,68 | 0,39 | 31 | 5 |
| broccoli | radioactive | atomic bomb | 0,97 | 0,76 | 8,02 | 5,90 | 3,19 | 0,50 | 33 | 0 | 0,98 | 0,73 | 10,38 | 7,79 | 3,08 | 0,26 | 40 | 0 |
| ironing board | wetsuit | surfboard | 0,93 | 0,78 | 6,62 | 5,48 | 2,72 | 0,40 | 27 | 0 | 1,00 | 0,82 | 4,56 | 3,54 | 1,96 | 0,11 | 28 | 0 |
| brush | lipstick | mascara | 1,00 | 0,72 | 9,04 | 5,46 | 3,38 | 0,44 | 32 | 1 | 0,92 | 0,59 | 8,87 | 7,74 | 2,56 | 0,28 | 39 | 0 |
| flatiron | salmon | ship | 0,83 | 0,43 | 14,80 | 17,20 | 4,60 | 0,64 | 30 | 0 | 0,86 | 0,43 | 9,73 | 12,22 | 4,29 | 0,46 | 28 | 0 |
| bullet | satellite | rocket | 0,93 | 0,81 | 6,70 | 5,93 | 2,84 | 0,40 | 27 | 0 | 0,96 | 0,71 | 8,75 | 5,93 | 2,37 | 0,33 | 28 | 0 |
| can | plunger | tiolet‡ | 0,97 | 0,70 | 8,04 | 8,27 | 2,59 | 0,28 | 33 | 0 | 0,94 | 0,42 | 11,16 | 9,22 | 3,07 | 0,31 | 31 | 0 |
| cat | pyramid | sphinx‡ | 0,94 | 0,71 | 9,97 | 6,29 | 2,75 | 0,34 | 34 | 0 | 0,97 | 0,31 | 9,76 | 6,88 | 2,94 | 0,35 | 32 | 0 |
| ceiling fan | conch | starfish | 0,89 | 0,58 | 8,16 | 6,40 | 2,94 | 0,31 | 36 | 0 | 0,90 | 0,62 | 9,44 | 8,76 | 3,23 | 0,40 | 39 | 2 |
| celery | swing | slide | 1,00 | 0,71 | 7,56 | 5,08 | 3,11 | 0,39 | 28 | 0 | 0,97 | 0,65 | 6,56 | 5,25 | 3,00 | 0,23 | 31 | 0 |
| cigarette | chalkboard | chalk | 1,00 | 0,97 | 4,29 | 3,70 | 2,55 | 0,38 | 29 | 0 | 1,00 | 0,87 | 3,45 | 3,19 | 2,00 | 0,33 | 30 | 0 |
| corset | stopwatch | hourglass | 0,95 | 0,77 | 10,00 | 9,25 | 2,29 | 0,24 | 22 | 0 | 0,84 | 0,47 | 11,22 | 7,81 | 3,70 | 0,37 | 32 | 0 |
| dalmatian | milk | cow | 0,96 | 0,64 | 9,47 | 6,95 | 3,11 | 0,48 | 28 | 0 | 0,97 | 0,57 | 10,30 | 6,20 | 2,52 | 0,21 | 30 | 0 |
| triangle* | notes | triangle | 0,97 | 0,79 | 8,45 | 4,94 | 2,86 | 0,43 | 29 | 1 | 0,97 | 0,81 | 7,82 | 6,93 | 1,84 | 0,29 | 32 | 0 |
| ferriswheel | wheat | water mill | 0,91 | 0,65 | 12,03 | 11,88 | 3,67 | 0,33 | 23 | 1 | 0,90 | 0,43 | 14,56 | 7,58 | 3,85 | 0,44 | 30 | 0 |
| flower | vinyl | phonograph | 0,93 | 0,70 | 10,52 | 9,81 | 3,16 | 0,28 | 27 | 1 | 0,92 | 0,69 | 12,42 | 10,78 | 3,71 | 0,38 | 26 | 0 |
| football | patch | scar | 0,90 | 0,34 | 14,48 | 15,07 | 4,22 | 0,68 | 41 | 0 | 0,88 | 0,25 | 14,74 | 13,24 | 4,43 | 0,43 | 32 | 8 |
| filmcan | circuit | battery | 0,97 | 0,73 | 8,10 | 6,36 | 2,83 | 0,45 | 30 | 0 | 0,97 | 0,46 | 9,84 | 5,38 | 3,97 | 0,47 | 37 | 0 |
| friedegg | tape | CD* | 0,85 | 0,63 | 12,68 | 9,70 | 3,65 | 0,57 | 27 | 5 | 0,97 | 0,70 | 12,95 | 11,84 | 2,97 | 0,31 | 30 | 12 |
| gasbottle | fire | fire extinguisher | 0,96 | 0,64 | 7,48 | 6,86 | 3,13 | 0,50 | 25 | 0 | 1,00 | 0,71 | 8,20 | 7,25 | 2,91 | 0,26 | 34 | 0 |
| watering can | giraffe | elephant | 0,86 | 0,62 | 13,98 | 10,21 | 3,92 | 0,48 | 29 | 0 | 0,97 | 0,58 | 14,02 | 10,88 | 3,33 | 0,40 | 31 | 1 |
| bell | scarf | hat | 0,88 | 0,77 | 9,56 | 9,00 | 4,04 | 0,57 | 26 | 0 | 0,82 | 0,43 | 11,83 | 6,45 | 3,57 | 0,43 | 28 | 0 |
| golf ball | astronaut | moon | 1,00 | 0,64 | 5,63 | 3,85 | 2,58 | 0,48 | 33 | 0 | 1,00 | 0,79 | 7,40 | 4,65 | 2,94 | 0,24 | 33 | 0 |
| grapes | party hat | balloons‡ | 0,91 | 0,66 | 7,33 | 4,34 | 2,63 | 0,34 | 35 | 0 | 0,92 | 0,50 | 12,29 | 10,41 | 3,03 | 0,33 | 36 | 0 |
| gun | comb | hairdryer‡ | 0,92 | 0,72 | 9,65 | 9,47 | 2,67 | 0,30 | 36 | 0 | 0,91 | 0,44 | 9,13 | 5,67 | 2,97 | 0,28 | 32 | 0 |
| handcuff | eye | glasses | 0,93 | 0,72 | 7,21 | 4,95 | 2,56 | 0,26 | 29 | 0 | 0,97 | 0,69 | 7,59 | 5,81 | 2,54 | 0,21 | 29 | 0 |
| phone | sliderule | calculator | 1,00 | 0,90 | 5,66 | 4,47 | 2,93 | 0,45 | 29 | 0 | 1,00 | 0,94 | 4,71 | 4,63 | 2,60 | 0,26 | 35 | 0 |
| hedgehog | barrette | brush‡ | 0,91 | 0,43 | 9,16 | 7,72 | 3,66 | 0,50 | 35 | 0 | 0,83 | 0,21 | 9,46 | 6,34 | 3,96 | 0,63 | 29 | 0 |
| ice cone | headphones | microphone | 0,96 | 0,79 | 6,12 | 6,01 | 2,52 | 0,37 | 28 | 0 | 0,91 | 0,67 | 9,21 | 4,37 | 2,60 | 0,17 | 33 | 0 |
| igloo | baseball | baseball cap | 0,80 | 0,03 | 21,17 | 7,72 | 4,57 | 0,71 | 35 | 0 | 0,88 | 0,15 | 13,83 | 11,46 | 3,86 | 0,45 | 33 | 0 |
| insect | car | helicopter | 0,96 | 0,46 | 11,10 | 6,56 | 3,48 | 0,52 | 26 | 0 | 0,97 | 0,56 | 9,95 | 5,22 | 2,71 | 0,29 | 32 | 0 |
| cable reel | filmtimer | film reel | 0,92 | 0,67 | 9,49 | 8,37 | 3,55 | 0,45 | 36 | 0 | 0,97 | 0,78 | 9,79 | 9,57 | 3,43 | 0,49 | 36 | 0 |
| necklace | dog | collar | 1,00 | 0,94 | 6,27 | 6,39 | 2,91 | 0,47 | 32 | 0 | 1,00 | 0,81 | 5,44 | 5,24 | 2,77 | 0,23 | 26 | 0 |
| button | hole | gully | 0,77 | 0,16 | 16,76 | 11,59 | 5,04 | 0,63 | 31 | 0 | 0,86 | 0,20 | 14,98 | 10,07 | 4,00 | 0,50 | 35 | 0 |
| tin | baby | crib | 0,96 | 0,79 | 10,08 | 9,45 | 4,13 | 0,48 | 24 | 2 | 0,97 | 0,85 | 8,21 | 7,25 | 2,82 | 0,24 | 34 | 1 |
| crab | nail1 | pliers‡ | 0,81 | 0,55 | 13,16 | 13,76 | 3,72 | 0,44 | 31 | 0 | 0,91 | 0,66 | 11,66 | 10,04 | 3,97 | 0,41 | 32 | 0 |
| ladder | loco | railtrack‡ | 0,90 | 0,45 | 13,69 | 10,17 | 3,46 | 0,31 | 29 | 0 | 0,85 | 0,38 | 14,78 | 10,75 | 3,73 | 0,41 | 26 | 0 |
| dragonfly | doll | fairy | 0,85 | 0,45 | 14,51 | 10,37 | 3,79 | 0,71 | 33 | 1 | 0,97 | 0,59 | 12,03 | 6,11 | 3,90 | 0,55 | 32 | 2 |
| linden leaf | card game | spade | 0,91 | 0,47 | 8,87 | 6,49 | 3,55 | 0,52 | 34 | 5 | 0,97 | 0,45 | 14,91 | 17,28 | 3,44 | 0,33 | 29 | 5 |
| ruler | pool | diving board | 0,89 | 0,49 | 7,53 | 3,40 | 3,23 | 0,45 | 35 | 0 | 0,95 | 0,42 | 12,46 | 7,25 | 3,44 | 0,39 | 38 | 0 |
| magnifier | pot | pan* | 0,97 | 0,68 | 7,28 | 6,11 | 2,60 | 0,30 | 31 | 0 | 0,97 | 0,80 | 6,54 | 3,58 | 2,09 | 0,15 | 35 | 10 |
| magnet | shoes | horseshoe | 0,87 | 0,39 | 9,65 | 9,02 | 3,81 | 0,44 | 31 | 0 | 0,93 | 0,15 | 15,66 | 9,27 | 3,40 | 0,36 | 27 | 0 |
| spiky ball | fish | blowfish | 0,95 | 0,54 | 7,29 | 4,89 | 3,03 | 0,43 | 37 | 6 | 0,97 | 0,74 | 8,26 | 5,29 | 2,82 | 0,21 | 35 | 6 |
| tape rule | frog | snail | 1,00 | 0,79 | 7,04 | 5,74 | 2,32 | 0,25 | 28 | 0 | 0,91 | 0,44 | 9,57 | 11,44 | 3,17 | 0,31 | 32 | 2 |
| microwave | oscar | TV | 0,87 | 0,63 | 17,29 | 15,58 | 4,42 | 0,54 | 30 | 0 | 0,92 | 0,59 | 11,18 | 8,13 | 3,50 | 0,41 | 37 | 1 |
| mosaic | poster | puzzle | 0,86 | 0,08 | 16,76 | 12,10 | 5,22 | 0,84 | 37 | 0 | 0,92 | 0,08 | 14,47 | 7,80 | 4,00 | 0,48 | 25 | 0 |
| marble | trampoline | rubber ball‡ | 0,96 | 0,54 | 12,41 | 12,98 | 4,28 | 0,52 | 26 | 0 | 0,93 | 0,30 | 13,46 | 18,96 | 4,96 | 0,79 | 30 | 0 |
| screwnut | bee | honeycomb | 0,97 | 0,75 | 10,03 | 6,62 | 3,19 | 0,52 | 32 | 0 | 1,00 | 0,79 | 11,29 | 10,16 | 3,04 | 0,46 | 28 | 0 |
| slug | coffee | croissant*‡ | 0,77 | 0,29 | 20,16 | 15,15 | 4,71 | 0,54 | 31 | 1 | 0,85 | 0,55 | 12,28 | 9,74 | 3,71 | 0,46 | 33 | 3 |
| hook | suction cup | octopus | 0,80 | 0,40 | 12,55 | 6,35 | 3,50 | 0,54 | 35 | 3 | 0,94 | 0,35 | 10,71 | 6,37 | 3,81 | 0,50 | 34 | 0 |
| folder | clef | accordion | 0,97 | 0,69 | 11,37 | 8,78 | 4,29 | 0,55 | 32 | 3 | 1,00 | 0,69 | 8,51 | 7,00 | 2,54 | 0,20 | 35 | 0 |
| paper pile | coin | banknote | 0,87 | 0,73 | 8,73 | 6,04 | 3,38 | 0,50 | 30 | 0 | 1,00 | 0,80 | 7,99 | 7,33 | 3,00 | 0,29 | 35 | 0 |
| pear | candle | light bulb | 0,92 | 0,65 | 12,31 | 10,33 | 3,46 | 0,38 | 26 | 0 | 0,81 | 0,33 | 17,01 | 12,07 | 3,93 | 0,55 | 36 | 0 |
| pen | drums | drumsticks‡ | 0,97 | 0,37 | 8,39 | 5,48 | 3,41 | 0,50 | 35 | 0 | 0,93 | 0,33 | 6,39 | 5,61 | 1,92 | 0,28 | 27 | 0 |
| penguin | cross | nun | 0,78 | 0,59 | 10,58 | 9,20 | 3,48 | 0,44 | 32 | 2 | 0,97 | 0,47 | 12,99 | 10,49 | 4,03 | 0,48 | 30 | 0 |
| peacock | ventilator | fan | 0,94 | 0,82 | 6,82 | 6,26 | 2,65 | 0,29 | 33 | 0 | 0,96 | 0,69 | 9,27 | 5,26 | 2,76 | 0,24 | 26 | 1 |
| mushroom | cagoule | umbrella | 0,84 | 0,51 | 9,53 | 6,85 | 3,29 | 0,65 | 37 | 0 | 1,00 | 0,50 | 9,51 | 7,76 | 3,41 | 0,44 | 32 | 1 |
| pistachio | pearls | clam | 0,92 | 0,54 | 12,68 | 11,14 | 3,42 | 0,42 | 26 | 2 | 0,97 | 0,72 | 10,14 | 8,51 | 3,64 | 0,39 | 29 | 6 |
| pocket watch | world map | compass | 0,79 | 0,46 | 12,99 | 11,70 | 4,11 | 0,53 | 24 | 0 | 0,87 | 0,43 | 14,60 | 13,25 | 3,85 | 0,42 | 30 | 0 |
| polaroid | mailbox | stamp‡ | 0,96 | 0,65 | 11,85 | 10,49 | 3,86 | 0,45 | 23 | 15 | 0,97 | 0,70 | 12,12 | 6,23 | 3,66 | 0,38 | 33 | 19 |
| porcupine | dustpan | broom | 0,93 | 0,68 | 8,84 | 7,35 | 3,73 | 0,54 | 28 | 0 | 0,97 | 0,71 | 8,76 | 6,66 | 3,27 | 0,33 | 34 | 0 |
| pumpkin* | trainers | basketball‡ | 0,88 | 0,50 | 11,04 | 7,04 | 2,78 | 0,52 | 26 | 0 | 0,94 | 0,32 | 9,18 | 6,70 | 3,75 | 0,47 | 34 | 0 |
| tire | hand | ring | 1,00 | 0,90 | 5,07 | 4,07 | 2,90 | 0,45 | 29 | 6 | 1,00 | 0,61 | 7,33 | 6,40 | 2,87 | 0,26 | 31 | 3 |
| cleanser | bird | swan* | 1,00 | 0,84 | 5,96 | 5,33 | 2,60 | 0,52 | 25 | 5 | 1,00 | 0,88 | 6,85 | 8,48 | 2,19 | 0,04 | 26 | 13 |
| lifesaver | muffin | doughnut | 1,00 | 0,84 | 6,78 | 4,62 | 2,41 | 0,25 | 32 | 0 | 1,00 | 0,81 | 6,37 | 5,54 | 2,76 | 0,22 | 37 | 1 |
| pipe system | mouse | maze | 0,92 | 0,49 | 13,32 | 11,58 | 4,09 | 0,59 | 37 | 0 | 1,00 | 0,59 | 9,40 | 6,23 | 3,65 | 0,41 | 37 | 0 |
| roots | blood | veins | 0,94 | 0,77 | 5,33 | 4,89 | 2,79 | 0,34 | 31 | 0 | 1,00 | 0,93 | 4,85 | 4,68 | 2,17 | 0,03 | 29 | 1 |
| salt shaker | racket | shuttlecock | 0,74 | 0,43 | 16,29 | 11,81 | 3,85 | 0,46 | 35 | 0 | 0,75 | 0,25 | 17,06 | 12,23 | 4,06 | 0,56 | 24 | 0 |
| box | motherboard | laptop | 0,96 | 0,72 | 9,54 | 7,93 | 3,21 | 0,50 | 25 | 0 | 1,00 | 0,71 | 8,81 | 7,05 | 2,82 | 0,29 | 38 | 0 |
| hose | poison | snake | 0,86 | 0,72 | 8,32 | 7,88 | 3,00 | 0,28 | 29 | 0 | 0,89 | 0,67 | 7,12 | 5,56 | 2,50 | 0,29 | 27 | 0 |
| sponge1 | cow | cheese | 0,97 | 0,67 | 8,37 | 5,90 | 2,97 | 0,34 | 30 | 0 | 0,95 | 0,53 | 9,09 | 2,42 | 3,17 | 0,39 | 19 | 2 |
| sponge2* | sharpener | eraser | 0,97 | 0,74 | 12,28 | 10,76 | 3,63 | 0,47 | 31 | 0 | 0,93 | 0,67 | 6,66 | 4,92 | 2,25 | 0,29 | 30 | 0 |
| soap | monitor | computer mouse | 0,97 | 0,83 | 4,77 | 5,02 | 2,89 | 0,21 | 29 | 0 | 0,92 | 0,42 | 6,14 | 4,21 | 2,33 | 0,17 | 26 | 0 |
| ski | noodles | chopsticks | 0,94 | 0,78 | 7,60 | 6,41 | 2,93 | 0,37 | 32 | 0 | 0,90 | 0,74 | 7,22 | 7,16 | 2,66 | 0,29 | 39 | 1 |
| sleeping bag | egypt | mummy* | 1,00 | 0,93 | 5,18 | 4,28 | 2,31 | 0,34 | 29 | 4 | 0,96 | 0,86 | 5,73 | 5,16 | 2,33 | 0,15 | 28 | 9 |
| snake | pants | belt | 0,90 | 0,73 | 6,74 | 5,17 | 2,85 | 0,37 | 30 | 0 | 0,97 | 0,76 | 5,42 | 3,59 | 2,16 | 0,25 | 33 | 3 |
| spade | cake | cake server‡ | 0,91 | 0,59 | 10,33 | 11,83 | 3,66 | 0,48 | 32 | 0 | 1,00 | 0,03 | 7,96 | 6,72 | 3,23 | 0,39 | 31 | 0 |
| coffee table | fly | spider | 0,87 | 0,57 | 10,26 | 7,65 | 3,85 | 0,62 | 30 | 0 | 1,00 | 0,77 | 11,40 | 12,04 | 3,94 | 0,39 | 31 | 0 |
| noodle | bottle opener | corkscrew | 1,00 | 0,81 | 8,44 | 8,53 | 2,96 | 0,23 | 26 | 0 | 1,00 | 0,57 | 11,46 | 11,04 | 3,67 | 0,30 | 30 | 0 |
| star fruit | saturn | star | 0,96 | 0,70 | 10,76 | 6,97 | 3,14 | 0,41 | 23 | 4 | 0,97 | 0,61 | 8,97 | 7,35 | 3,05 | 0,41 | 38 | 5 |
| socket | face mask | face | 0,92 | 0,58 | 8,84 | 6,26 | 3,00 | 0,33 | 26 | 0 | 1,00 | 0,39 | 11,21 | 7,64 | 3,61 | 0,42 | 33 | 0 |
| pin | candy | lollipop | 0,83 | 0,79 | 4,71 | 4,57 | 1,70 | 0,10 | 24 | 0 | 1,00 | 0,87 | 4,37 | 4,55 | 2,31 | 0,21 | 39 | 0 |
| stamp | nail2 | hammer | 0,88 | 0,75 | 9,50 | 8,26 | 3,90 | 0,43 | 24 | 0 | 0,96 | 0,78 | 10,97 | 8,92 | 3,73 | 0,42 | 27 | 0 |
| stone | stethoscope | heart | 1,00 | 0,92 | 3,34 | 3,15 | 2,21 | 0,41 | 39 | 0 | 1,00 | 0,94 | 3,21 | 3,04 | 1,67 | 0,15 | 33 | 0 |
| street light | crown | scepter | 0,96 | 0,72 | 8,25 | 7,19 | 2,83 | 0,67 | 25 | 0 | 0,90 | 0,50 | 11,51 | 9,99 | 3,33 | 0,41 | 30 | 8 |
| match | burger | fries | 0,92 | 0,72 | 4,84 | 3,32 | 2,00 | 0,17 | 25 | 0 | 1,00 | 0,69 | 7,32 | 5,38 | 2,88 | 0,28 | 32 | 0 |
| T | sweatpants | t-shirt | 0,92 | 0,81 | 9,66 | 8,12 | 2,92 | 0,54 | 26 | 1 | 0,94 | 0,63 | 11,07 | 8,73 | 3,03 | 0,23 | 32 | 2 |
| lamp | blanket | tent | 1,00 | 0,88 | 6,56 | 6,10 | 2,53 | 0,34 | 32 | 0 | 1,00 | 0,85 | 4,37 | 3,87 | 2,33 | 0,12 | 33 | 0 |
| tiefighter | weight bench | barbell | 0,92 | 0,76 | 8,94 | 8,40 | 2,76 | 0,21 | 37 | 0 | 0,97 | 0,62 | 9,84 | 7,24 | 3,00 | 0,36 | 29 | 0 |
| whiteout | mousetrap | mouse | 0,96 | 0,88 | 5,69 | 4,69 | 2,50 | 0,33 | 25 | 0 | 0,97 | 0,61 | 14,73 | 14,39 | 4,10 | 0,53 | 31 | 0 |
| paddle | crosswalk | stop sign | 0,97 | 0,61 | 7,39 | 6,43 | 3,56 | 0,38 | 33 | 3 | 1,00 | 0,85 | 6,51 | 5,40 | 2,21 | 0,21 | 33 | 0 |
| evil eye perl | binoculars | eye | 1,00 | 0,77 | 8,19 | 4,10 | 2,77 | 0,55 | 22 | 3 | 1,00 | 0,74 | 7,21 | 5,35 | 3,13 | 0,32 | 31 | 2 |
| tower | flashlight | light house | 0,91 | 0,35 | 12,22 | 8,32 | 3,67 | 0,60 | 34 | 0 | 1,00 | 0,36 | 11,11 | 9,35 | 3,57 | 0,43 | 28 | 0 |
| tv tower | pills | syringe | 1,00 | 0,88 | 5,53 | 5,25 | 2,36 | 0,21 | 33 | 0 | 0,97 | 0,89 | 5,56 | 4,50 | 2,17 | 0,23 | 36 | 2 |
| tyrannosaur | baby sling | kangaroo | 0,66 | 0,13 | 15,51 | 7,34 | 4,52 | 0,48 | 32 | 0 | 0,83 | 0,41 | 9,54 | 7,15 | 3,79 | 0,38 | 29 | 0 |
| ufo | hockey helmet | puck | 0,92 | 0,33 | 12,51 | 15,16 | 4,55 | 0,58 | 36 | 0 | 0,93 | 0,27 | 12,31 | 18,18 | 3,14 | 0,39 | 30 | 0 |
| spatula | mosquito | fly swatter | 1,00 | 0,94 | 4,95 | 4,66 | 2,65 | 0,32 | 31 | 0 | 0,97 | 0,83 | 7,90 | 5,81 | 2,57 | 0,37 | 36 | 0 |
| vase | coffin | urn | 0,97 | 0,86 | 7,43 | 5,61 | 2,79 | 0,50 | 29 | 0 | 0,97 | 0,62 | 10,95 | 7,27 | 2,94 | 0,28 | 37 | 5 |
| power strip | turn sign | traffic lights | 0,94 | 0,69 | 9,83 | 6,54 | 3,58 | 0,42 | 35 | 0 | 0,93 | 0,53 | 10,86 | 5,53 | 3,75 | 0,39 | 30 | 0 |
| birdcage | gavel | prison | 0,97 | 0,77 | 10,21 | 8,59 | 3,17 | 0,40 | 31 | 0 | 0,90 | 0,84 | 7,73 | 7,20 | 2,50 | 0,18 | 31 | 0 |
| scale | playground | seesaw | 1,00 | 0,73 | 4,59 | 4,52 | 2,37 | 0,07 | 30 | 0 | 0,97 | 0,50 | 9,83 | 6,35 | 3,15 | 0,39 | 34 | 0 |
| walnut | skeleton | brain‡ | 1,00 | 0,76 | 4,04 | 2,23 | 2,12 | 0,33 | 33 | 1 | 0,96 | 0,93 | 3,66 | 4,18 | 1,81 | 0,04 | 27 | 5 |
| helmet | chameleon | turtle | 0,88 | 0,42 | 13,60 | 8,04 | 3,83 | 0,48 | 33 | 0 | 1,00 | 0,32 | 8,54 | 10,83 | 3,21 | 0,32 | 28 | 0 |
| washer | tripod | camera | 0,97 | 0,73 | 7,93 | 7,63 | 2,97 | 0,47 | 33 | 0 | 1,00 | 0,87 | 7,04 | 6,82 | 2,65 | 0,19 | 31 | 0 |
| batting | sugar shaker | cotton candy | 0,89 | 0,14 | 10,32 | 6,69 | 4,00 | 0,56 | 28 | 0 | 0,93 | 0,27 | 10,52 | 10,91 | 3,32 | 0,36 | 30 | 1 |
| wheel | oven | pizza | 0,77 | 0,38 | 12,07 | 8,57 | 3,60 | 0,45 | 26 | 0 | 0,93 | 0,52 | 12,04 | 6,95 | 3,19 | 0,30 | 29 | 0 |
| zebra | road | crosswalk | 1,00 | 0,93 | 6,59 | 6,52 | 2,19 | 0,30 | 27 | 0 | 0,90 | 0,67 | 6,81 | 5,95 | 3,33 | 0,41 | 30 | 0 |
| sugar cane | wheelchair | cane | 0,97 | 0,81 | 6,69 | 6,07 | 3,19 | 0,45 | 32 | 0 | 0,93 | 0,80 | 6,83 | 6,21 | 2,73 | 0,11 | 40 | 0 |
| onion dome | carrot | onion* | 0,87 | 0,70 | 9,22 | 7,14 | 2,77 | 0,35 | 30 | 3 | 0,92 | 0,62 | 12,18 | 6,14 | 3,50 | 0,44 | 37 | 9 |

*Note*. Button press = correctly and incorrectly solved items; RT = solution time (in seconds); RT if correct = solution time when solution was correct (in seconds); AHA = AHA! experience in %; AHA: Suddenness = Suddenness of the solution (part of AHA! experience) on a scale from 0 (continuous) to 6 (very sudden); N = amount of participants; alternative solutions = sum of correctly named alternative solutions; N with altern. sol. = amount of subjects with correct alternative solution; the asterisk (or #) in the *LI-RAT solution* column represents items that had 3 or 4 correct alternative solutions in Spanish (or German) sample. The double dagger (‡) in the solution column represents a difference bigger than 1.5SD from the mean accuracy difference between the Spanish and English sample; the asterisk in the *LI-RAT perceptual cue* column represents items that differ more than 2SD from the mean suddenness difference between the Spanish and English sample. The correspondent pictures to the cues are freely available online here: github.com/MaxiBecker/LI-RAT.git.

**Table S3**

*Influence of sample, verbal semantic fluency and demographics on LI-RAT performance (accuracy, solution time) and AHA! experience (including suddenness).*

|  |  |  |  |  |  |  |
| --- | --- | --- | --- | --- | --- | --- |
|  | **Accuracy** | | | **Solution time** | | |
| Predictors | OR | CI | p | ß | CI | p |
| (Intercept) | 1.96 | 1.72 – 2.23 | 0.281 | 1.10 | 1.04 – 1.16 | <0.001 |
| sample [German] | 1.14 | 0.87 – 1.48 | 0.336 | -0.01 | -0.14 – 0.11 | 0.780 |
| sample [Spanish] | 0.84 | 0.67 – 1.05 | 0.120 | 0.08 | -0.02 – 0.19 | 0.128 |
| word_fluency | 1.19 | 1.09 – 1.29 | **<0.001** | -0.12 | -0.16 – -0.08 | **<0.001** |
| age | 1.00 | 0.91 – 1.10 | 0.975 | 0.07 | 0.02 – 0.11 | **0.004** |
| gender [female] | 0.94 | 0.80 – 1.11 | 0.490 | 0.03 | -0.05 – 0.11 | 0.432 |
| R² | 0.091 |  |  | 0.156 |  |  |
|  |  |  |  |  |  |  |
|  | **AHA! experience** | | | **Suddenness** | | |
| Predictors | OR | CI | p | ß | CI | p |
| (Intercept) | 1.00 | 0.83 – 1.19 | 0.218 | 0.03 | -0.18 – 0.23 | <0.001 |
| sample [German] | 1.39 | 0.97 – 2.00 | 0.075 | 0.31 | -0.10 – 0.73 | 0.136 |
| sample [Spanish] | 2.08 | 1.51 – 2.85 | **<0.001** | 0.29 | -0.06 – 0.65 | 0.105 |
| word_fluency | 1.05 | 0.94 – 1.18 | 0.409 | 0.07 | -0.05 – 0.20 | 0.257 |
| age | 1.08 | 0.94 – 1.23 | 0.290 | 0.01 | -0.14 – 0.16 | 0.883 |
| gender [female] | 1.00 | 0.80 – 1.26 | 0.998 | -0.27 | -0.53 – -0.01 | **0.040** |
| R² | 0.089 |  |  | 0.032 |  |  |

*Note*. OR = Odds Ratio; CI= 95% confidence interval; *p*= p-Value; ß = standardized beta estimates.

***Figure S7.***

*Histogram of AHA experience and suddenness for raw values in study 1*


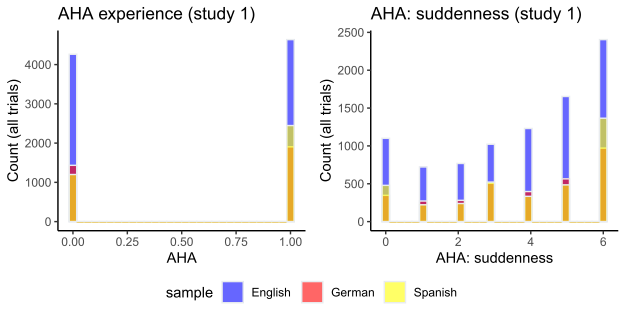

Supplement: Supplementary file 1 — (DOCX 161 kb) [file 13428_2021_1773_MOESM1_ESM.docx]
